# *De novo* Assembly of Transcriptomes From a B73 Maize Line Introgressed With a QTL for Resistance to Gray Leaf Spot Disease Reveals a Candidate Allele of a Lectin Receptor-Like Kinase

**DOI:** 10.3389/fpls.2020.00191

**Published:** 2020-03-13

**Authors:** Tanya Welgemoed, Rian Pierneef, Lieven Sterck, Yves Van de Peer, Velushka Swart, Kevin Daniel Scheepers, Dave K. Berger

**Affiliations:** ^1^Centre for Bioinformatics and Computational Biology, University of Pretoria, Pretoria, South Africa; ^2^Department of Biochemistry, Genetics and Microbiology, University of Pretoria, Pretoria, South Africa; ^3^Forestry and Agricultural Biotechnology Institute, University of Pretoria, Pretoria, South Africa; ^4^Department of Plant Biotechnology and Bioinformatics, Ghent University, Ghent, Belgium; ^5^Department of Plant Systems Biology, VIB, Ghent, Belgium; ^6^Genomics Research Institute, University of Pretoria, Pretoria, South Africa; ^7^Department of Plant and Soil Sciences, University of Pretoria, Pretoria, South Africa

**Keywords:** maize, *Cercospora*, gray leaf spot, QDR, lectin receptor-like kinase, *de novo*, transcriptome

## Abstract

Gray leaf spot (GLS) disease in maize, caused by the fungus *Cercospora zeina*, is a threat to maize production globally. Understanding the molecular basis for quantitative resistance to GLS is therefore important for food security. We developed a *de novo* assembly pipeline to identify candidate maize resistance genes. Near-isogenic maize lines with and without a QTL for GLS resistance on chromosome 10 from inbred CML444 were produced in the inbred B73 background. The B73-QTL line showed a 20% reduction in GLS disease symptoms compared to B73 in the field (*p* = 0.01). B73-QTL leaf samples from this field experiment conducted under GLS disease pressure were RNA sequenced. The reads that did not map to the B73 or *C. zeina* genomes were expected to contain novel defense genes and were *de novo* assembled. A total of 141 protein-coding sequences with B73-like or plant annotations were identified from the B73-QTL plants exposed to *C. zeina*. To determine whether candidate gene expression was induced by *C. zeina*, the RNAseq reads from *C. zeina*-challenged and control leaves were mapped to a master assembly of all of the B73-QTL reads, and differential gene expression analysis was conducted. Combining results from both bioinformatics approaches led to the identification of a likely candidate gene, which was a novel allele of a lectin receptor-like kinase named L-RLK-CML that (i) was induced by *C. zeina*, (ii) was positioned in the QTL region, and (iii) had functional domains for pathogen perception and defense signal transduction. The 817AA L-RLK-CML protein had 53 amino acid differences from its 818AA counterpart in B73. A second “B73-like” allele of L-RLK was expressed at a low level in B73-QTL. Gene copy-specific RT-qPCR confirmed that the *l-rlk-cml* transcript was the major product induced four-fold by *C. zeina*. Several other expressed defense-related candidates were identified, including a wall-associated kinase, two glutathione s-transferases, a chitinase, a glucan beta-glucosidase, a plasmodesmata callose-binding protein, several other receptor-like kinases, and components of calcium signaling, vesicular trafficking, and ethylene biosynthesis. This work presents a bioinformatics protocol for gene discovery from *de novo* assembled transcriptomes and identifies candidate quantitative resistance genes.

## Introduction

Maize is one of the three main cereal crops that are globally used as a food source and is especially important in regions such as Africa, where it is a staple food (Ranum et al., 2014). It contributes significantly to food security and is a widespread crop in smallholder farming, but its production is often hampered in developing countries by various factors including diseases (Shiferaw et al., 2011).

A recent global survey highlighted several fungal foliar diseases that reduce maize yields significantly in Africa, Asia, and the Americas (Savary et al., 2019). One of these foliar diseases is gray leaf spot (GLS), which is present throughout sub-Saharan Africa, where GLS is caused by *Cercospora zeina* (Meisel et al., 2009; Nsibo et al., 2019). GLS can also be caused by *Cercospora zeae-maydis*, which is the predominant pathogen in the United States; consequently, most studies to date have focused on disease-resistance mechanisms against this fungal species (Benson et al., 2015; Yang et al., 2017).

Methods aimed at controlling GLS in large-scale maize production include tillage, crop rotation, fungicide application, and cultivating resistant hybrids (Latterell and Rossi, 1983; Ward et al., 1999; Mallowa et al., 2015). Tillage can be effective in regions where there is a fallow/winter period between plantings, since inoculum is buried. However, tillage has negative environmental effects on soil and moisture conservation, and this approach is less effective in many parts of sub-Saharan Africa where maize is grown continuously, providing a constant source of inoculum. Small-holder farmers often cannot afford or prefer to avoid chemical control, and therefore planting disease-resistant maize is the most desirable strategy (Nsibo et al., 2019).

Breeding for resistance for foliar diseases such as GLS is therefore one of the major goals of crop protection. Maize resistance to GLS in germplasm tested globally has indicated that this trait is quantitative in nature, since many genetic mapping studies have identified QTL for GLS resistance (Berger et al., 2014). This is in contrast to some foliar diseases of maize, such as northern leaf blight (NLB) and southern rust, against which maize exhibits both quantitative and qualitative resistance (Jines et al., 2007; Chung et al., 2011; Hurni et al., 2015).

Meta-analysis of QTL for disease resistance in maize has identified a large number of QTL across the maize genome (Wisser et al., 2006), and, for GLS, there were seven QTL “hotspots” across the genome based on a comparison of a dozen prior studies (Berger et al., 2014). Quantitative disease resistance (QDR) is a term used to describe resistance conferred by multiple loci such as QTL (Nelson et al., 2018). Plant breeders have long recognized that deploying QDR is more durable than a single qualitative major resistance gene (Nelson et al., 2018). This is because a major resistance gene results in a high selection pressure for the pathogen to overcome its effect. The challenge for plant breeders, though, is that individual QTL loci have a small effect on phenotypic variation and may be influenced by the genetic background.

One approach to working with the complexity of QDR is to identify DNA markers linked to the QTL that can be used for marker-assisted breeding. Genome-wide association studies (GWAS) have been deployed to identify SNPs associated with QDR, such as a glutathione-*S*-transferase linked to resistance to GLS, NLB, and SCLB (Wisser et al., 2011). Combining QTL mapping with GWAS was used to efficiently locate SNPs that were tightly linked to QTL for GLS resistance, which can subsequently be deployed for marker-assisted breeding (Benson et al., 2015; Mammadov et al., 2015).

However, the ultimate marker is within the causal gene (French et al., 2016). The most common route to identifying causal genes is to start with a QTL of major effect and carry out fine-mapping to narrow down the genomic region to the causal gene. This is a laborious process, but it has been successful in defining some QTL for maize disease resistance. QDR to head smut in maize was conferred by a wall-associated kinase (Zuo et al., 2014), and QDR to GLS and southern corn leaf blight was narrowed down to a novel allele of a lignin biosynthesis gene (Yang et al., 2017). However, in some cases, this approach only narrows down the search to a genomic region with several candidate genes; for example, QDR to GLS at a locus on chromosome five was fine-mapped to a region with 15 genes (Xu et al., 2014). QDR to GLS on chromosome eight derived from teosinte was fine-mapped to a region with five genes (Zhang et al., 2017). In another study, association mapping was used to identify QTL for GLS resistance (Benson et al., 2015). One QTL was found to be associated with intervein distance on the maize leaf, and another was fine-mapped to a region with several genes, one of which encoded a detoxification enzyme; however, the causative gene was not identified.

Another approach for identifying candidate genes underlying a QTL is to combine QTL mapping, expression QTL (eQTL) analysis, and co-expression analysis on a population segregating for disease resistance (Christie et al., 2017). This was applied to a maize population segregating for GLS resistance, and due to sampling at the peak of GLS disease, revealed a co-expression network that was highly correlated with susceptibility (Christie et al., 2017). The co-expression network was a set of 179 genes that were co-expressed at a higher level in GLS-susceptible plants of a RIL population. The network was significantly enriched for genes with trans-eQTLs that coincided on the genetic map with two phenotypic QTL alleles in the susceptible phase. This approach also identified a co-expression network that correlated with resistance, which highlighted a potential role for jasmonate signaling in resistance. Anti-microbial kauralexin production was shown to be induced by *C. zeina* infection, and contrasting levels of kauralexins were found in resistant and susceptible RILs from the mapping population (Meyer et al., 2017).

The genome sequence of maize line B73 (Jiao et al., 2017) and other inbred lines has greatly facilitated the identification of DNA markers and candidate genes for QDR. However, a limitation is that the most effective alleles or genes conferring QDR may be derived from germplasm that is genetically distinct from B73. A comparison of 19 maize inbred lines and 14 teosinte genotypes against the B73 reference found 3210 genes that are missing or have a lower copy number than the B73 reference (Swanson-Wagner et al., 2010). Another study found at least 2000 genes between the B73 and Mo17 maize lines that differ in copy number or presence/absence (Springer et al., 2009).

Further evidence for diversity of gene content in maize germplasm has come from RNAseq data. Three studies conducted RNAseq on diverse maize lines and identified novel (non-B73) transcripts by mapping the reads to the B73 genome, collecting the unmapped reads, and assembling these *de novo* into transcripts. These studies identified 157, 1321, and 2355 novel transcripts from 6, 21, and 368 diverse inbred maize lines, respectively (Lai et al., 2010; Hansey et al., 2012; Jin et al., 2016). These findings imply that comparison to the few sequenced genomes of maize may not facilitate the identification of novel genes or alleles responsible for some cases of QDR.

This provides the rationale for this study in which a transcriptomics approach is used to contrast gene expression between near-isogenic lines in the B73 background that differ only in the QTL locus from a diverse maize source. The hypothesis is that transcripts derived from B73 will be common to both NILs and that these can be discarded after mapping reads to the high-quality single-molecule sequenced B73 genome. In addition, the genome sequence of the pathogen *C. zeina* is also available, so the reads can be simultaneously mapped to this genome, allowing removal of the fungal reads. The reads that are left are potentially derived from the QTL, and these can be *de novo* assembled and annotated to identify candidate genes.

In this study, we applied this approach to a GLS QTL on chromosome 10 that we had previously identified in maize inbred line CML444 (Berger et al., 2014). Near-isogenic lines were created in the B73 background using marker-assisted backcross breeding. RNAseq was carried out on these NILs that were exposed to *C. zeina*, and unmapped reads were *de novo* assembled. We also conducted differential gene expression analysis on control and *C. zeina*-challenged leaf material. Our approach led to the identification of an allele of a lectin receptor-like kinase from CML444 positioned in the QTL region and induced by the pathogen as the most likely candidate gene, as well as a range of other defense-related transcripts that could contribute to the QDR to GLS disease.

## Materials and Methods

### Plant Material

The maize material used in this study was comprised of near-isogenic lines in the inbred B73 background. A previous QTL mapping study of a RIL population derived from the parents CML444 and SC Malawi identified a GLS disease severity QTL on chromosome 10 (Berger et al., 2014). In that study, the QTL region was named 10G2_GLS and 10H_GLS from two field trials in KwaZulu-Natal province, South Africa. The CML444 allele was associated with GLS resistance and will be named QTL10 here. In this study, QTL10 was backcrossed from CML444 into the GLS susceptible B73 line to produce the near-isogenic line B73 + QTL10 (B73-QTL). During the backcross breeding, SSR markers were used for foreground selection [two markers flanking the QTL region (umc1084 and bnlg1450) and two markers located within the QTL region (umc1196 and umc11760) (Berger et al., 2014)] (Supplementary Figure S1). Background selection to select against the CML444 background was carried out using a panel of proprietary SNP markers (data not shown). The final NILs were in the BC3S2 generation and were estimated to contain only 6.8% CML444 genomic content corresponding to the QTL10 region.

### GLS Disease Assessment of Field-Grown Maize

The B73 and B73-QTL NILs were planted at Greytown, KwaZulu-Natal, South Africa in a randomized block design with three replicate blocks of ten plants per row each. This site is a hotspot for GLS disease. GLS disease and RNAseq data from the B73 plants in this field trial have been reported by Christie et al. (2017) and Swart et al. (2017) (NCBI GEO accession GSE94442; RRID:SCR_006472); however, the current study used data from the B73-QTL plants.

The GLS disease severity of the B73 and B73-QTL maize plants was assessed using a 1–9 scale as reported in Berger et al. (2014) at five time points from 66 to 103 dap in the field trial at Greytown. Data were collected separately from each of the three biological replicate rows of each genotype, and the area under the disease progress curve (AUDPC) was calculated for each replicate.

GLS disease severity was also assessed using digital image analysis to calculate disease lesion area, and the amount of *C. zeina* gDNA in the same leaf samples was quantified by quantitative PCR (qPCR) as a proxy for the fungal biomass, using the methods described in Korsman et al. (2012). Leaf samples were collected at 77 dap at the VT stage of development, at which stage the susceptible B73 plants had developed GLS lesions below the ear but had very few lesions above the ear, as described in Christie et al. (2017). Leaf samples (10 cm × 10 cm) collected above the ear were named “control” samples, whereas leaf samples collected below the ear were “*C. zeina*-challenged” samples. It was expected that the lower leaves would be subjected to greater *C. zeina* challenge than the upper leaves, on account of epidemiological knowledge of the disease that inoculum is mainly derived from maize stubble from the previous season (Ward et al., 1999). However, the upper leaves were not expected to be devoid of *C. zeina*. Three biological replicates of each treatment (B73 control, B73-QTL control, B73 *C. zeina*-challenged, B73-QTL *C. zeina*-challenged) were collected, with upper and lower leaf samples taken from the same plant. Two leaves per treatment were sampled to make up a biological replicate; for example, B73-QTL control “replicate one” was made up of a pool of two upper leaf pieces from B73-QTL plant 1, and two lower leaves of the same plant were sampled and pooled for the B73-QTL *C. zeina*-challenged “replicate one.” The statistical significance of the differences between B73 and B73-QTL disease severity data was determined by Student’s *t*-tests at a 95% confidence interval in R (R Project for Statistical Computing, RRID:SCR_001905).

### RNA Sequencing of Field-Grown Maize

The same B73-QTL leaf samples from the field trial carried out at Greytown, South Africa described above and used for GLS disease severity assessment were processed for RNA sequencing. The sub-sampling to quantify *C. zeina* gDNA by qPCR and RNA extraction methods have been described for the B73 samples in Christie et al. (2017), and the B73-QTL samples were processed at the same time in the same way. In brief, total RNA was extracted using Qiazol, treated with DNAse, and purified with an RNeasy Plant Mini kit according to the manufacturer’s instructions (Qiagen, Hilden, Germany). RNA sequencing was done as described in Christie et al. (2017) by BGI Tech Solutions Co., Ltd. (Beijing Genome Institute, Hong Kong; RRID:SCR_011114) on an Illumina HiSeqTM 2000 instrument with 200 bp insert libraries and a 100 bp paired-end module (Illumina Inc., San Diego, CA, United States, RRID:SCR_010233). This resulted in six datasets of RNA-seq reads made up of three replicates of the B73-QTL control and three replicates of the B73-QTL *C. zeina*-challenged leaves. The B73 RNAseq dataset has been reported in Christie et al. (2017).

### Bioinformatics Strategy to Identify Candidate Genes Within the QTL Region

**FIGURE 1:**
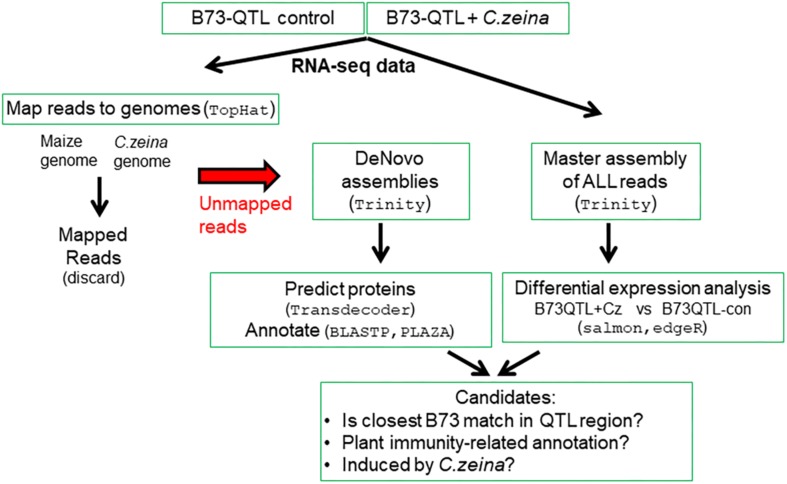
Bioinformatics strategy for identifying candidate genes within the QTL region.

The bioinformatics pipeline involved the following main steps: (i) identifying RNAseq reads that did not map to the maize or fungal genomes; (ii) *de novo* assembly of the unmapped reads into transcripts; (iii) annotation of the *de novo* assembled transcripts; and (iv) differential expression analysis. The last step involved constructing a “master assembly” of all plant and fungal reads from a pool of all six B73-QTL samples to provide a reference to which reads in each replicate could be mapped, followed by differential gene expression analysis (Figure 1).

The B73-QTL RNAseq FASTQ read files were examined with FastQC (RRID:SCR_014583) (Andrews, 2016) to identify adaptor sequences and low-quality reads and nucleotides. Strict quality filtering was used to trim the reads using Trimmomatic (RRID:SCR_011848) (Bolger et al., 2014). Lower-quality reads would be less likely to map to the reference genomes, so it was important to remove these reads to ensure that only good-quality reads were retained in the pool of unmapped reads for the *de novo* assembly. Trimmomatic produces four FASTQ output files for each set of forward and reverse FASTQ read files: a paired forward file and a paired reverse file, and an unpaired forward and an unpaired reverse file. Only the paired forward and reverse files were used for further analysis.

### Bioinformatics Pipeline 1: *de novo* Assembly of Unmapped Reads

The filtered B73-QTL RNA seq reads were mapped simultaneously to a combined “maize/Cz reference sequence” of both maize B73 and *C. zeina* CMW25467 genome sequences. Unmapped reads were identified from the B73-QTL control separately from the B73-QTL *C. zeina*-challenged samples by pooling the RNAseq reads from the three replicates of each treatment prior to mapping to the “maize/Cz reference sequence.” TopHat2 (TopHat, RRID:SCR_013035) (Kim et al., 2013) with default settings was used to map the RNAseq reads to the B73 v.4 reference genome ftp://ftp.ensemblgenomes.org/pub/plants/release-34/fasta/zea_mays/ (Jiao et al., 2017) and the *C. zeina* draft genome (GenBank accession MVDW01000014.1). TopHat 2 was chosen for the mapping since it was designed to map reads to the main transcripts as well as splice variants (Kim et al., 2013), thus ensuring a high likelihood that all reads derived from B73 transcripts would be mapped, leaving only novel reads for the subsequent *de novo* assembly.

The unmapped reads produced by TopHat2 were converted to FASTA format using the FASTX-toolkit (Gordon and Hannon, 2010) and were *de novo* assembled using Trinity (RRID:SCR_013048) (Haas et al., 2013). First, the Trinity parameters for *de novo* assembly were optimized using only the reads that mapped to the B73 genome (Supplementary Table S1). The optimal Trinity *de novo* assembly parameters used for assembly of the unmapped reads from the B73-QTL samples were the default parameters with k-mer size = 31.

The open reading frames in each of the two Trinity FASTA files (B73-QTL control and B73-QTL *C. zeina*-challenged) were determined using TransDecoder (Haas et al., 2013), which was developed for use with Trinity. First, it identifies all the possible open reading frames above a specified length (in this case, 50 amino acids) in each transcript. Second, it predicts and translates the most likely protein from each transcript. The likelihood is determined through a log-likelihood score based on the nucleotide position in the codon and relative frequency in transcript sequences. TransDecoder produced FASTA output files of (i) all possible coding sequences, (ii) the most likely coding sequences, and (iii) their amino acid translations. The predicted protein sequences were annotated by comparing them to the NCBI (RRID:SCR_006472) nr protein database using BLASTp with an e-value cut-off of 0.01.

### Bioinformatics Pipeline 2: Differential Expression Analysis

The main aim was to determine if any of the novel transcripts from the unmapped RNAseq reads were differentially expressed between control B73-QTL and *C. zeina*-challenged B73-QTL leaf samples. It should be noted that, since these samples were taken from upper (termed “control”) and lower (termed “*C. zeina*-challenged”) leaves of the same plants, there are three potential factors that could result in differential expression of a gene: (i) induction by *C. zeina*, (ii) developmental differences between upper and lower leaves, or (iii) systemic responses in the upper leaves from the *C. zeina*-challenged lower leaves.

The two separate *de novo* assemblies could not be compared directly, since not all transcripts were assembled fully in both *de novo* assemblies. Therefore, a *de novo* “master assembly” was compiled from all reads from the experiment to serve as a reference for mapping and counting the reads from each biological replicate. Trinity (with the same parameters as described above) was used to compile the “master assembly” from a pool of the quality-filtered RNAseq reads from the three B73-QTL control replicates and the three B73-QTL *C. zeina*-challenged samples.

Subsequently, the reads from each of the six samples were then mapped separately to the “master assembly” and quantified using Salmon (RRID:SCR_017036) software (Patro et al., 2017). Salmon is a computationally fast transcriptome-wide quantifier that accounts for GC content bias and has high accuracy. The output from Salmon of read counts per transcript in the master assembly in each of the six datasets was compiled using the abundance_estimates_to_matrix.pl perl script in the Trinity utilities (Haas et al., 2013). The transcript counts were evaluated for significant differences between the B73-QTL *C. zeina*-challenged and control conditions using the R package edgeR (RRID:SCR_012802) (Robinson et al., 2010). The trimmed mean of M-values were applied for normalization in edgeR. The false discovery rate (FDR) values were determined from the *p*-values using the Benjamini–Hochberg method (Robinson and Oshlack, 2010).

The functional annotations of the differentially expressed genes (FDR < 0.05) were determined by comparing them to the B73 v.4 (Jiao et al., 2017) and *C. zeina* (Wingfield et al., 2017) reference genomes using BLAT (RRID:SCR_011919) (Kent, 2002). Transcripts were classified as B73 or *C. zeina* genes if they had at least 80% identity across 90% of the transcript’s length, which was determined using a custom AWK script. The fungal genes were discarded. The remaining differentially expressed transcripts that did not match B73 or *C. zeina* were searched against the nr database using BLASTx to identify those with similarity to plant genes.

Transcripts classified as either B73 or with similarity to plant genes were uploaded into the Monocots PLAZA 4.0 workbench (with B73 v4 annotations)^1^ (Van Bel et al., 2018) for annotation. During the import process, the sequences were compared to the PLAZA maize B73 v4 database using BLASTn and the information on the closest match retrieved. Transcripts with more than 95% nucleotide identity to B73 genes were excluded as originating from the B73 background of B73-QTL. Transcripts that had matched other plants according to the BLASTx search against nr were also verified against the PLAZA database entries for the same plant and the nr BLASTx protein annotation used. The output was a set of variants of B73 transcripts that were potential novel alleles (named B73-like) or transcripts of “novel” maize genes absent in B73 but present in CML444.

Another aim of the differential gene expression analysis was to compare global gene expression of maize genes between B73 and B73-QTL plants challenged with *C. zeina*. A previous study (Swart et al., 2017) had examined differential expression in B73 control and B73 *C. zeina*-challenged plants in the same field trial (GEO database GSE94442). This data was compared to the expression profiles of B73 genes in B73-QTL plants that were found to be differentially expressed in response to *C. zeina*. GO enrichment analysis of the B73-QTL differentially expressed genes was carried out in PLAZA 4.0 using the hypergeometric distribution, and the Bonferroni method was applied to correct for multiple testing. RNAseq data for the six replicates of B73-QTL have been deposited in the GEO database (GSE137198).

### Combining Data From *de novo* Assembly and Differential Gene Expression to Identify Candidate Genes

The results of the two different analyses to identify QTL candidate genes in the B73-QTL plants, namely (i) *de novo* assembly of unmapped reads, and (ii) differential expression analysis after mapping to a master assembly, were combined at follows.

Transcripts assembled *de novo* from the unmapped reads were matched to the transcripts in the master assembly by BLASTn of the B73-QTL master assembly transcripts against the B73-QTL control and B73-QTL *C. zeina*-challenged unmapped and *de novo* assembled transcripts. Matches with over 90% identity across over 90% of the query length were defined as the same transcript in the two datasets. Finally, a single combined table was compiled that listed the B73-QTL transcripts with B73-like or plant annotations in the following categories: (i) master assembly transcripts that were differentially expressed, and (ii) transcripts from the *de novo* assemblies of unmapped reads. All the bioinformatics scripts have been deposited at https://zenodo.org/record/3384940. This repository also includes the FASTA files of all the B73-QTL *de novo* assembled transcripts and predicted proteins listed in the combined table.

### Construction of Full-Length Transcript of the Candidate Lectin Receptor-Like Kinase Gene

The transcripts from *de novo* assembly of unmapped reads and the master assembly from B73-QTL that matched the lectin receptor-like kinase gene encoded by B73 Zm00001d026382 were combined. Sequence and annotation information of Zm00001d026382 was obtained from B73 RefGen_v4 at MaizeGDB^2^ (RRID:SCR_006600) (Portwood et al., 2018). Since reads that mapped to B73 Zm00001d026382 would have been excluded from the “unmapped reads assembly,” an additional step was carried out. All reads from B73-QTL were mapped to Zm00001d026382 alone, and matching reads were collected and *de novo* assembled. These transcripts were then combined with those from the unmapped reads and master assembly of B73-QTL to obtain the full-length transcript of the gene from B73-QTL.

### PCR-RFLP and Sequencing

The PCR-RFLP assay to distinguish between the alleles of the lectin receptor-like kinase gene in B73 and CML444 was designed from an alignment of the B73 Zm00001d026382 gDNA sequence and the corresponding *de novo*-assembled transcript from B73-QTL. CLC Main Workbench 8.0.1 (CLC Main Workbench, RRID:SCR_000354) and Primer Designer (Sci Ed Software, Denver, CO, United States) were used to design the assay. PCR was conducted with the primers Lectin-F and Lectin-R (Supplementary Table S2) to amplify a 430 bp fragment within exon 1 of Zm00001d026382 that contained polymorphic restriction enzyme sites (*Bam*HI and *Hph*I). PCR amplification conditions were: 3 min at 95°C, followed by 30 cycles of 30 s at 95°C, 30 s at 55°C, and 30 s at 72°C, and a final extension step of 5 min at 72°C. Sanger sequencing of the PCR products, restriction enzyme digestions, and visualization of the products by agarose gel electrophoresis were carried out using standard protocols.

### Synteny Analysis

Genome sequences of other maize lines and the wild relative teosinte available on MaizeGDB and sorghum available on NCBI (GCF_000003195.3) were queried with the B73-QTL *de novo* assembled transcript of the lectin receptor-like kinase (variant of B73 Zm00001d026382) using BLASTn to identify putative syntelogs. Some of the genomes were not annotated, so regions with positive hits were manually annotated by identification of open reading frames using CLC Main Workbench. A synteny diagram was constructed with iADHoRe (within PLAZA) between genomic regions containing the putative syntelog of the B73-QTL lectin receptor-like kinase and several upstream and downstream gene models.

### Maize Inoculations With *C. zeina* in the Glasshouse

The maize glasshouse inoculation experiment with *C. zeina* isolate CMW25467 was conducted using the protocol described in Swart et al. (2017). Maize B73 and B73-QTL plants were inoculated at the V8 stage of development (7 weeks after planting). An area of 100 cm^2^ on leaf four from the base of each maize plant was marked and inoculated with 1 × 10^6^ *C. zeina* conidia per milliliter using a soft paintbrush on replicate plants. Mock-inoculated plants were treated with water and kept in a separate section of the glasshouse. At 43 days post-inoculation (dpi), the maize plants were at the R1 stage of development, and mature GLS lesions were observed on the susceptible B73 plants. At this time point, all marked leaf samples were collected and flash-frozen in liquid nitrogen and stored at −80 prior to RNA extraction. Four biological replicate plants of each treatment were sampled, i.e., *C. zeina*-inoculated B73, *C. zeina*-inoculated B73-QTL, and mock-inoculated plants of both genotypes.

### RT-PCR and RT-qPCR Analysis

Maize RNA was extracted from the B73 and B73-QTL leaf samples from the glasshouse trial treatments using QIAzol lysis reagent (Qiagen, Hilden, Germany) according to the manufacturer’s instructions. A TURBO DNA-free kit (Thermo Fisher Scientific, Waltham, MA, United States) was used to remove any contaminating genomic DNA. cDNA synthesis was carried out with a Maxima H Minus First Strand cDNA Synthesis Kit (Thermo Fisher Scientific, Waltham, MA, United States) following the manufacturer’s instructions. Each reverse transcriptase reaction contained 2 μg RNA. RT-PCR was conducted with a primer pair in exon 4 and exon 7 regions that were identical between the CML444 and B73 (Zm00001d026382) full-length L-RLK transcripts (L-RLK-qFor e4 primer; L-RLK-qRev e7 primer) (Supplementary Table S2). An intron-flanking banana actin primer pair (Van den Berg et al., 2004) (Supplementary Table S2) was used to confirm the absence of contaminating gDNA in the cDNA samples.

RT-qPCR was performed according to the MIQE guidelines. Primer Designer 4 (Sci Ed Software, Raleigh, NC, United States) was used to design primers. Three sets of primers were designed to distinguish expression between the CML and B73 copy of the L-RLK in B73-QTL plants and to profile the fully spliced variants. First, primers in the conserved exon 7 were used to measure the expression of both gene copies together (L-RLK e7 F1 and L-RLK e7 R1) (Supplementary Table S2). Second, sequence differences in the 5′ end of exon 5 between the CML and B73 gene copies were used to design a CML-specific F1 exon-exon primer (L-RLK e4e5 F1 [cml]) and a B73-specific exon-exon F2 primer (L-RLK e4e5 F2 [b73]). Each F primer matched the 3′ end of exon 4 and 5′ end of exon 5, so they could only amplify transcripts resulting from removal of the intron between exon 4 and exon 5. The reverse primers within exon 5 were designed to a region with a 3′ end SNP between the CML and B73 copies for added specificity (L-RLK e5 R1 [cml] and L-RLK e5 R2 [b73]). Primer sequences are given in Supplementary Table S2, and their positions on the B73 L-RLK gene model are shown with a diagram and alignments in Supplementary Figures S2a,b, respectively.

Primer specificity was tested using conventional PCR, assessing the melting curves, and sequencing the RT-qPCR products. RPOL (DNA-directed RNA polymerase – Zm00001d012857) was used as a reference gene, using primers developed for normalization by Ma et al. (2006) and previously used for this purpose in a B73-GLS glasshouse experiment (Christie et al., 2017). RT-qPCR was carried out on four biological replicates of each treatment with three technical replicates of each biological replicate using a Bio-Rad CFX96TM Real-Time PCR detection system (Bio-Rad, California, United States). Each amplification reaction was set up in a total volume of 8 μl that consisted of the following: 1 × SsoAdvanced^TM^ Universal SYBR^®^ Green Supermix (Bio-Rad, California, United States), 10 μM of each primer, 100 ng of template cDNA, and sterile distilled water. The PCR cycling conditions were as follows: 95°C for 30 s, followed by 35 cycles of 95°C for 15 s and 60°C for 30 s. RT-qPCR data were analyzed using qbase + software, version 3.0 (Biogazelle, Zwijnaarde, Belgium)^3^. Statistical significance was evaluated by one-way ANOVA with a Tukey-Kramer *post hoc* test in qbase + software.

## Results

### Field Resistance to GLS Was Conferred by QTL10 in the B73 Background

**FIGURE 2:**
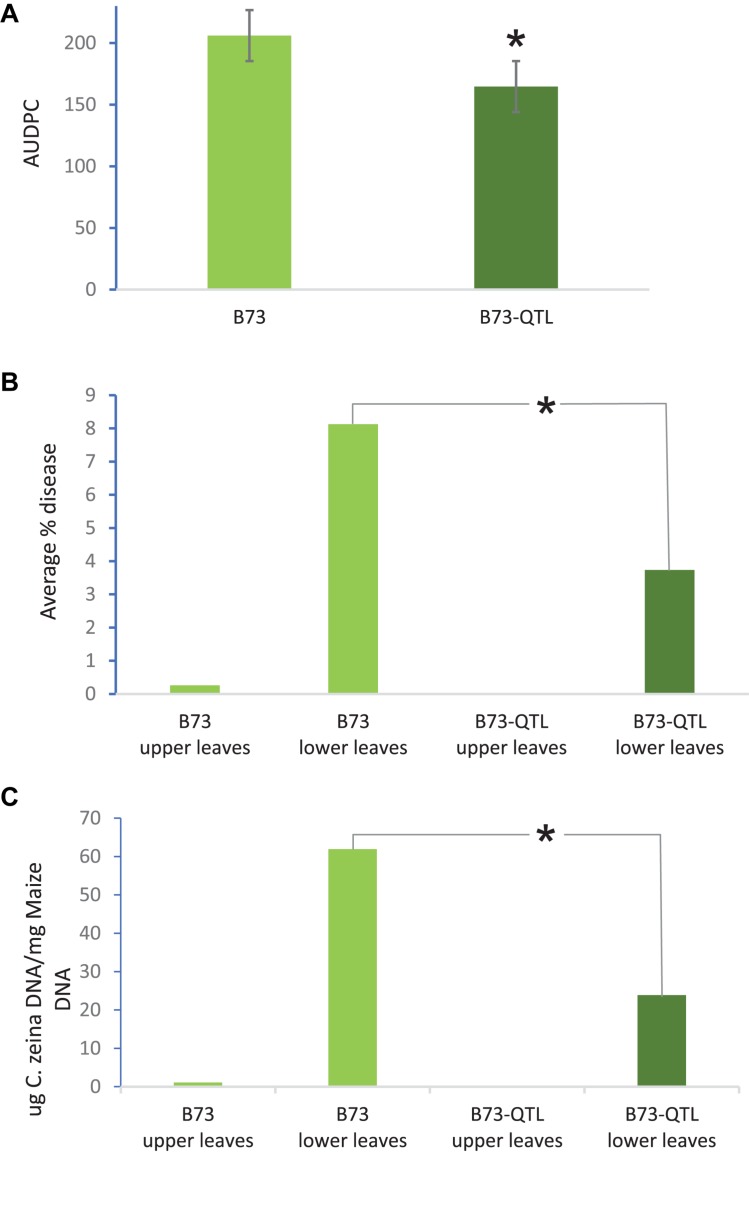
B73-QTL plants have lower GLS disease severity in the field than B73 plants. **(A)** Area under disease progress curve of GLS scores for B73 and B73-QTL maize lines. **(B)** Quantification of *C. zeina* gDNA content (as a proxy for fungal load) between B73 and B73-QTL upper and lower leaves. Fungal load was below the threshold for B73-QTL upper leaves. **(C)** Quantification of GLS disease lesion area between B73 and B73-QTL upper and lower leaves by digital image analysis. Lesion areas were below the quantification threshold for B73-QTL upper leaves. All panels: *significant difference (Student’s *t*-test, *p* < 0.05) between B73 lower leaves and B73-QTL lower leaves. Error bars represent standard errors.

QTL10 from the maize inbred line CML444 had previously been shown to account for 20% of the phenotypic variation in a mapping population of the parents CML444 and SC Malawi (Berger et al., 2014). In the current study, GLS severity scores showed that B73-QTL plants showed a 20% reduction in disease compared to B73 plants in the field trial at Greytown, South Africa (*p* = 0.01, Figure 2a). These AUDPC values were assessed over the peak of GLS disease pressure during flowering (66–103 dap). GLS disease severity was also assessed in the leaf samples collected for RNA sequencing at 77 dap. At this time point, only a few immature lesions were visible in leaves above the ear in B73 plants, whereas leaves below the ear had developed mature lesions (Christie et al., 2017). Under field conditions, the lower leaves are infected first from *C. zeina* inoculum on debris in the soil, and the pathogen progresses via rain splash upward through several infection cycles as the plant matures (Meisel et al., 2009). Digital image analysis indicated that B73-QTL had an average GLS lesion area on lower leaves of 3.7% compared to 8.1% on B73 lower leaves, a reduction of 54% (*p* < 0.05) (Figure 2b). *C. zeina* fungal load measured by qPCR corroborated this result, showing a 61% reduction in *C. zeina* gDNA in B73-QTL leaf samples compared to B73 (*p* < 0.05) (Figure 2c).

### *De novo* Assembly and Annotation of Transcripts From Reads That Did Not Map to the B73 or *C. zeina* Genomes

RNA sequencing of the B73-QTL control and B73-QTL *C. zeina-*challenged leaf samples produced 24–28 million reads per biological replicate (Supplementary Table S3). Subsequent read trimming and filtering, which included removal of orphan reads without a read pair, produced a dataset of 8–11 million read pairs per sample, which equated to 1.2–1.6 Gb of sequence data per sample for downstream analysis (Supplementary Table S3). RNAseq data from the three replicates of the B73-QTL control and B73-QTL *C. zeina-*challenged leaf samples were pooled separately and mapped to a combined “reference genome” of maize B73 and *C. zeina* (Figure 1). As expected, most of the reads mapped to this reference, namely 96% and 92% of the B73-QTL control and B73-QTL *C. zeina-*challenged reads, respectively (Table 1). Approximately 1 and 2 million read pairs from the B73-QTL control and B73-QTL *C. zeina-*challenged pools of reads, respectively, did not map to the “reference genome” of B73/*C. zeina*, and these were retained for *de novo* assembly of transcripts (Table 1).

**TABLE 1:** B73-QTL RNAseq read mapping and *de novo* assembly results.

	B73-QTL control	B73-QTL *C. zeina-*challenged
Read pairs in each pool of three replicates	29,256,535	25,704,511
Read pairs mapped to “reference genome” of B73 and *C. zeina*	28,182,260 (96%)	23,577,640 (92%)
Read pairs that did not map	1,074,256 (4%)	2,126,871 (8%)
*De novo* assembled transcripts from unmapped reads	927	5938
Proteins predicted from the *de novo* assembled transcripts (total)	676	4403
Plant proteins*	122 (89)	95 (68)
Fungal proteins*	235 (209)	3952 (2819)
Other proteins*	63 (58)	92 (88)
Unidentified proteins*	256	264

*Values in brackets give numbers of non-redundant proteins of each annotation type.

The parameters for *de novo* assembly using the software Trinity were first optimized using the set of reads that mapped to the B73 genome (Supplementary Table S1), and these parameters were applied to the two sets of unmapped reads. The B73-QTL control sample produced 927 transcripts from the unmapped reads, and 676 proteins greater than 50 AA were predicted from this dataset using Transdecoder (Table 1). As expected, due to its higher fungal load, the B73-QTL *C. zeina*-challenged sample yielded a larger number of *de novo*-assembled transcripts from the unmapped reads (5938) (Table 1). A total of 4403 proteins were predicted from this sample. BLASTP analysis showed that the majority of these proteins identified from unmapped reads were of fungal origin. This is most likely due to the fact that the *C. zeina* draft genome is less complete and not as well annotated as the B73 genome (Jiao et al., 2017).

There was a total of 300 transcripts from both datasets that had BLASTP hits to proteins of maize or plant origin (Supplementary Table S4), some of which corresponded to the same protein. This produced a set of 141 non-redundant plant proteins, with 89 and 68 from the control and *C. zeina*-challenged samples, respectively, with 16 present in both (Table 1 and Supplementary Table S4). This set contained 19 proteins involved in defense, signaling, or stress responses (Supplementary Table S4). The remainder had the following functional annotations: transcription (3), translation (8), metabolism (21), actin/ubiquitin (12), and unknown (81). The next step was to determine whether any of these were differentially expressed in response to challenge with *C. zeina*, and individual gene candidates will therefore be discussed in the next section.

### Differential Expression Analysis Between B73-QTL Control and *C. zeina*-Challenged Leaves

There were two goals of the differential expression analysis: first, to identify patterns of global gene expression in *C. zeina*-challenged B73-QTL plants, and second, to determine whether any of the potentially novel *de novo* assembled transcripts were differentially expressed in response to *C. zeina*. The GLS lesion area and *C. zeina* qPCR data (Figures 2b,c) showed that the fungal load in the upper “control” leaves was below the limit of detection, in contrast to the “*C. zeina*-challenged” lower leaves. Therefore, differential expression of a particular gene could be due to *C. zeina* infection, but it could also be due to developmental differences between upper and lower leaves or systemic responses from lower leaves.

The strategy followed was to construct a single transcript “master assembly” from all B73-QTL samples and then map the reads from each replicate to the master assembly and conduct differential expression analysis. After *de novo* assembly of all the reads, the transcript master assembly consisted of 57,800 transcripts (>200 nucleotides in size each) (data not shown). The differential expression analysis identified which transcripts were induced or repressed in the B73-QTL *C. zeina-*challenged leaves compared with the control. All transcripts in the master assembly were annotated in three categories – B73 genes, B73-like genes, “plant” genes, fungal genes, and “other” genes. A total of 5236 B73 and B73-like maize genes, 47 plant genes, 209 fungal genes, and 100 other genes were found to be significantly differentially expressed (FDR < 0.05).

**FIGURE 3:**
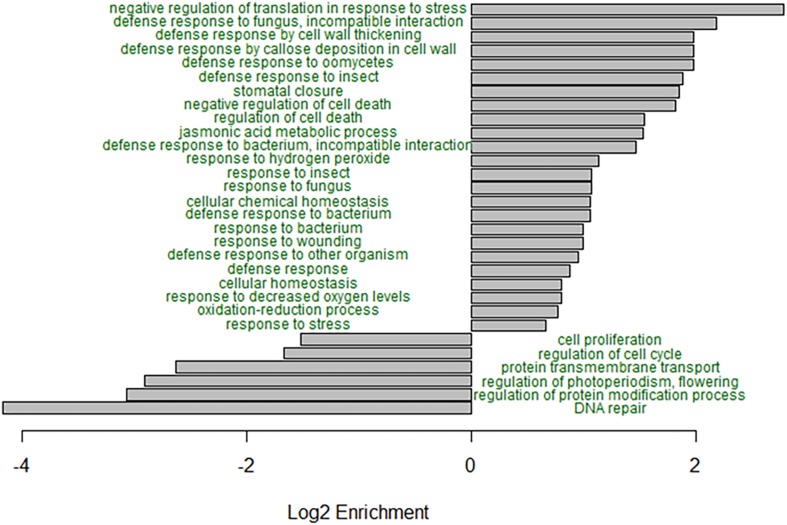
GO enrichment analysis of the differentially expressed genes in B73-QTL between control and *C. zeina*-challenged leaves. GO enrichment analysis was conducted in PLAZA 4.0 with the dataset of 5492 differentially expressed B73-QTL master assembly transcripts with B73, B73-like, or plant annotations. The significance of over- or under-representation of GO terms compared to the maize B73 proteome was determined using the hypergeometric distribution, and the Bonferroni method was applied to correct for multiple testing. Log2 enrichment for selected enriched or depleted biological process terms is plotted. All GO terms shown had an adjusted *p*-value < 0.05.

GO enrichment analysis of the B73, B73-like, and plant genes that had higher expression in the *C. zeina-*challenged leaves showed overrepresentation of the GO terms related to responses to biotic stress (Figure 3). Notably, the GO terms “defense response to fungus, incompatible interaction,” “defense response by cell wall thickening,” “defense response by callose deposition in cell wall,” “stomatal closure,” “negative regulation of cell death,” “jasmonic acid metabolic process,” “response to hydrogen peroxide,” and “negative regulation of translation in response to stress” were enriched in the *C. zeina-*challenged leaves, as would be expected in a leaf stressed by fungal invasion (Figure 3). The under-represented GO terms included “DNA repair,” “regulation of protein modification process,” “regulation of photoperiodism, flowering,” and “cell proliferation,” as would be expected in a leaf focusing less on growth (Figure 3).

The B73-QTL transcripts in the master assembly that were annotated as B73-like or plant genes were next extracted from the results of the differential expression analysis. This showed that out of 346 transcripts, 46% were more highly expressed in the *C. zeina*-challenged leaves than in control leaves, and 54% were repressed (Supplementary Table S4). The B73-QTL transcripts identified from the *de novo* assemblies of the unmapped reads as encoding potentially novel maize proteins were then matched to the transcripts in the master assembly and combined into a single dataset of 547 transcripts encoding 377 non-redundant proteins (Supplementary Table S4). The transcripts were annotated based on the most similar protein in maize B73 if a match could be found.

### Identification of Candidate Genes

The next step was to manually filter the gene list from both analyses (Supplementary Table S4) to identify candidates for the QTL10 effect based on whether: (i) the closest matching B73 gene was positioned in the QTL10 region, (ii) the annotation was associated with plant immunity, and/or (iii) a candidate’s expression was higher in lower leaves where *C. zeina* fungal load was greater. Selected candidate genes are shown in Table 2.

**TABLE 2:** Selected candidate genes from the B73-QTL transcripts (master assembly and/or *de novo* assembly of unmapped reads).

Assembled transcript (B73-QTL)^1^	Dataset (B73-QTL)^2^	Closest match description^3^	Functional Annotation^4^	Closest match (B73 gene)^5^	Closest match (Protein)^6^	Chr^7^	B73 Cz/Con^8^	B73-QTL Cz/Con^9^
DN12415_c0_g1_i1	MADN	G-type lectin S-receptor-like serine/threonine-protein kinase B120	Defense/Signaling	Zm00001d026382	AQK46433	Chr10-QTL	ns	UP
DN3882_c0_g1_i1| m.15432	DN	G-type lectin S-receptor-like serine/threonine-protein kinase B120	Defense/Signaling	Zm00001d026382	AQK46433	Chr10-QTL	ns	nt
DN4163_c0_g1_i1| m.14842	DN	G-type lectin S-receptor-like serine/threonine-protein kinase B120	Defense/Signaling	Zm00001d026382	AQK46433	Chr10-QTL	ns	nt
DN868_c0_g1_i1| m.628	DN	G-type lectin S-receptor-like serine/threonine-protein kinase B120	Defense/Signaling	Zm00001d026382	AQK46433	Chr10-QTL	ns	nt
DN554_c0_g1_i1| m.144	DN	1-aminocyclopropane-1-carboxylate oxidase	Defense	Zm00001d024258	XP_020400957	Chr10	n/a	nt
DN12960_c0_g1_i2	MA	Chitinase	Defense	Zm00001d036370	ACJ62113*	Chr06	nt	UP
DN33263_c0_g1_i1	MA	Exocyst component SEC6	Defense	Zm00001d018060	AQK74955	Chr05	ns	UP
DN8155_c0_g1_i1	MA	Glucan endo-1,3-beta-glucosidase 13	Defense	Zm00001d021695	ONM58041	Chr07	ns	UP
DN902_c0_g1_i1	MA	lipid-transfer protein	Defense	Zm00001d014513	AQK66449	Chr05	ns	UP
DN10018_c0_g1_i1	MA	Plasmodesmata callose-binding protein 3	Defense	Zm00001d013250	AQK62762	Chr05	nt	UP
DN3435_c0_g1_i1	MA	Remorin family protein	Defense	Zm00001d020329	ONM54720	Chr07	ns	DOWN
DN3838_c0_g1_i2	MA	Ca-dependent lipid-binding protein	Defense/Signaling	Zm00001d019750	ONM53685	Chr07	UP	UP
DN12633_c0_g1_i2	MA	Calcineurin B-like protein	Defense/Signaling	Zm00001d029976	ONL99914	Chr01	ns	UP
DN10536_c0_g2_i2	MA	Calmodulin	Defense/Signaling	Zm00001d047597	AQL07073	Chr09	ns	UP
DN11063_c0_g1_i2	MA	Calmodulin	Defense/Signaling	Zm00001d038543	AQK86889.1	Chr06	nt	UP
DN1943_c0_g1_i1	MA	GTP binding protein	Defense/Signaling	Zm00001d025660	AQK44198	Chr10	ns	UP
DN13488_c0_g1_i2	MA	G-type lectin S-receptor-like serine/threonine-protein kinase LECRK4	Defense/Signaling	Zm00001d035949	AQK79998	Chr06	DOWN	UP
DN5447_c0_g1_i1| m.13539	DN	LRR receptor-like protein kinase	Defense/Signaling	Zm00001d026303	AQK46158	Chr10	ns	nt
DN9303_c0_g1_i2	MA	LRR receptor-like serine/threonine-protein kinase	Defense/Signaling	Zm00001d051083	AQK53592	Chr04	ns	UP
DN40593_c0_g1_i1	MA	Receptor-like protein kinase	Defense/Signaling	Zm00001d003670	ONM17453	Chr02	DOWN	UP
DN681_c0_g1_i1| m.2245	DN	Receptor-like serine/threonine-protein kinase SD1-8	Defense/Signaling	Zm00001d002199	ONM13390	Chr02	nt	nt
DN12860_c0_g1_i3	MADN	Tetraspanin family protein	Defense/Signaling	Zm00001d030014	NP_001150827	Chr01	nt	UP
DN13662_c2_g2_i2	MA	Wall-associated receptor kinase 5	Defense/Signaling	Zm00001d036261	AQK80511	Chr06	ns	UP
DN603_c0_g1_i1| m.13313	DN	Abscisic stress ripening protein 1	Defense/Stress	Zm00001d025401	ACG35620	Chr10	UP	nt
DN8769_c0_g1_i2	MADN	Abscisic stress ripening protein 5	Defense/Stress	Zm00001d025401	AQK43681	Chr10	UP	UP
DN3012_c0_g1_i1| m.12378	DN	Glutathione S-transferase 20	Defense/Stress	Zm00001d043795	ONM39050.1	Chr03	UP	nt
DN4081_c0_g1_i1	MADN	Glutathione S-transferase GSTU6	Defense/Stress	Zm00001d043795	ONM39050	Chr03	UP	UP
DN14045_c1_g1_i7	MA	NAD(P)-linked oxidoreductase	Metabolism	Zm00001d025528	AQK43905	Chr10	ns	UP
DN34410_c0_g1_i1	MA	WRKY transcription factor	Transcription	Zm00001d026252	AQK45916	Chr10	UP	UP

^1^Transcript assembled *de novo* from B73-QTL RNAseq reads (note that names have the prefix “TRINITY_” in Supplementary Table S3). ^2^Transcript source, either (i) MA = Master Assembly of all B73-QTL reads, (ii) DN = *de novo* assembly of unmapped B73-QTL reads, or (iii) MADN = transcripts with the same annotation present in both datasets. ^3^Description of closest protein match in maize inbred line B73 identified by BLASTX of Master assembly transcripts or BLASTP of proteins predicted from the unmapped reads (BLAST scores and E-values are provided in Table S3). ^4^Functional annotation of closest match. ^5^B73 gene model of closest match (B73 v4 annotation). ^6^NCBI accession of closest protein match (*Zea mays*). ^7^Chromosome location of closest B73 match (Chr10-QTL indicates that the location is within the QTL10 region). Chromosome positions are provided in Supplementary Table S3. ^8^Differential expression of closest B73 match in B73 plants (Cz infected/control): UP = fold change > 1, FDR < 0.05; DOWN = fold change < 1, FDR < 0.05; ns = FDR > 0.05; nt = not expressed). Data from Christie et al., 2017. ^9^Differential expression of master assembly transcript in B73-QTL plants (Cz infected/control): UP = fold change > 1, FDR < 0.05; DOWN = fold change < 1, FDR < 0.05; ns = FDR > 0.05; nt = not tested since differential expression analysis not done on unmapped transcripts). *Closest match was to protein from (*Zea mays subsp. parviglumis*).

Only one candidate gene from B73-QTL had its closest match to a B73 gene that was situated within the QTL10 region (Table 2). This candidate was represented by several assembled transcripts from B73-QTL, and the match was to a G-type lectin S-receptor-like serine/threonine-protein kinase B120 (Table 2). It will be discussed in detail in the next section. First, candidates with interesting defense-related annotations will be reported.

Table 2 lists three genes with annotations similar to genes associated with quantitative resistance to maize diseases, namely a wall-associated kinase (Zuo et al., 2014), two glutathione s-transferases (Wisser et al., 2011), and a remorin (Jamann et al., 2016). The wall-associated kinase and one of the glutathione s-transferases were expressed higher in the *C. zeina*-challenged lower leaves in the current study. Three PR proteins, namely a chitinase, a glucan beta-glucosidase, and a lipid transfer protein were increased by three, four, and ten-fold in lower leaves compared to upper leaves, respectively (Table 2 and Supplementary Table S4). Another candidate from the *de novo* assembly was 1-aminocyclopropane-1-carboxylate oxidase, the rate-limiting step in ethylene biosynthesis (Houben and Van de Poel, 2019). Components of vesicular trafficking (a tetraspanin and an exocyst protein), and cell wall protection (plasmodesmata callose-binding protein) were expressed higher in lower leaves that had greater *C. zeina* content. Calcium signaling candidates were also expressed higher in lower leaves, namely a calcineurin, two calmodulins, and a calcium-dependent lipid-binding protein. The short-list also included five other receptor-like kinases and two abscisic stress ripening proteins (Table 2). Most of the listed candidates were differentially expressed in B73-QTL, but their closest match in B73 was not (Table 2 and Supplementary Table S4).

### Candidate Gene Encoding a Maize Lectin Receptor-Like Kinase

Three different transcripts from the B73-QTL unmapped *de novo* assemblies encoded proteins that were similar to parts of the same G-type lectin S-receptor-like serine/threonine-protein kinase B120 (AQK46433) from B73 (Table 2 and Supplementary Table S4). Furthermore, the corresponding transcript in the master assembly of B73-QTL was expressed two-fold higher in lower leaves compared to upper leaves, indicating that it might be induced by *C. zeina* (Table 2 and Supplementary Table S4). The B73 gene model for this protein is Zm00001d026382 (B73 RefGen_v4) or Zm00001e041728 (B73 RefGen_v5), and it is positioned on chromosome 10 in a region corresponding to QTL10 from CML444.

A full length 2811 nt transcript was constructed from the B73-QTL *de novo* assemblies (Acc# MT108451), and this was predicted to encode an 817 AA protein (designated L-RLK-CML in this study; Acc# QIH29483) with 93.5% identity to the 818 AA protein from B73 (designated B73 v5 L-RLK P2 in this study; B73 v4 accession: XP_008661637; B73 v5 accession: Zm00001e041728_P002) (Supplementary Figure S3a). L-RLK-CML and B73 v5 L-RLK P2 both contain (i) the extracellular domains D-mannose binding lectin, S-locus glycoprotein, and PAN-like, (ii) a trans-membrane domain, and (iii) an intracellular tyrosine kinase domain with an active site signature (Supplementary Figure S3b and Supplementary Table S5).

Evidence for expression of the full-length *l-rlk-cml* transcript in B73-QTL was verified by inspection of the RNAseq reads that mapped to the Zm00001d026382 genomic DNA sequence. High coverage was observed across all seven predicted exons encoding the 818AA protein B73 v5 L-RLK P2 (Supplementary Figure S3d). Furthermore, RNAseq reads from CML444 challenged with *C. zeina* in a separate field experiment showed high coverage across the 2811 nt *l-rlk-cml* transcript assembled from the B73-QTL reads (Supplementary Figure S3e).

### CML444 and B73-QTL Plants Have Two Copies of the Lectin Receptor-Like Kinase Gene

**FIGURE 4:**
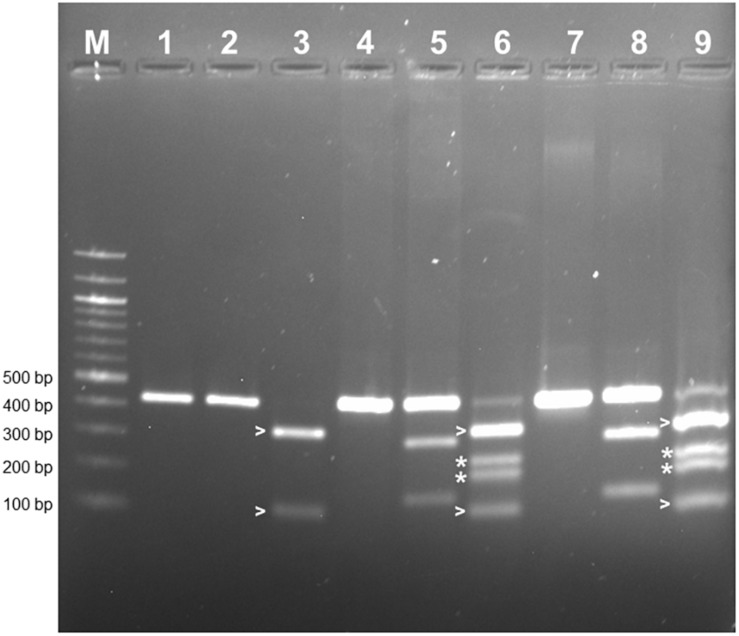
PCR-RFLP to distinguish alleles of the lectin receptor kinase in B73, CML444, and B73-QTL gDNA. The PCR product amplified from maize gDNA with the Lectin F and R primers corresponded to a 430 bp fragment in Exon 1 of B73 gene Zm00001d026382. The 430 bp product amplified from each genotype was either digested with *Bam*HI or *Hph*I. Lanes 1–3: B73 uncut, *Bam*HI, *Hph*I, respectively; Lanes 4–6: CML444 uncut, *Bam*HI, *Hph*I, respectively; Lanes 7–9: B73-QTL uncut, *Bam*HI, *Hph*I, respectively. The B73 allele lacks a *Bam*HI site (lanes 2, 5, and 8). The *Bam*HI digestion products of the CML444 allele are 296 and 134 bp in size (lanes 5 and 8). The HpHI digestion products (324 and 106 bp) of the B73 allele are marked with white arrowheads (lanes 3, 6, and 9). The HpHI digestion products (234 and 196 bp) of the CML444 allele are marked with white asterisks (lanes 6 and 9). Lane M = 100 bp DNA Ladder (New England Biolabs).

PCR-RFLP was employed to confirm that the allele encoding L-RLK-CML was present in CML444 and B73-QTL and absent in B73. A 430 bp fragment within exon 1 of the B73 gene Zm00001d026382 was predicted to contain a *Hph*I site but no *Bam*HI site, whereas the corresponding fragment in *l-rlk-cml* had a *Hph*I site at a different position but contained a *Bam*HI site (restriction enzyme maps shown in Supplementary Figure S4a). *Bam*HI did not digest the 430 bp PCR product from B73 gDNA (Figure 4, lane 2), whereas it cut the product from CML444 and B73-QTL gDNA into 296 bp and 134 bp fragments, as expected (Figure 4, lanes 5 and 8). However, a significant proportion of the CML444 and B73-QTL PCR products remained undigested by *Bam*HI. To determine whether this was due to partial digestion or the presence of a B73-like allele in CML444, a different restriction enzyme was used for PCR-RFLP. *Hph*I digestion of the 430 bp PCR product from B73 gDNA produced diagnostic fragments of 324 and 106 bp (Figure 4, lane 3 and Supplementary Figure S4a), whereas *Hph*I digestion of the 430 bp product from CML444 or B73-QTL produced different fragments of 234 and 196 bp, as expected (Figure 4, lanes 6 and 9 and Supplementary Figure S4a). In addition, the CML444 or B73-QTL samples also produced the B73-like fragments of 234 and 196 bp (Figure 4, lanes 6 and 9). These results indicated that CML444 and B73-QTL have two copies of the lectin receptor-like kinase: one that corresponds to the transcript encoding L-RLK-CML and one that is B73-like. DNA sequencing of the 430 bp PCR product from B73 and B73-QTL confirmed the positions of the *Bam*HI and *Hph*I restriction enzymes and showed that the B73-QTL product was a mixture of the two alleles as shown by double peaks at the *Bam*HI and *Hph*I restriction sites (Supplementary Figure S4b).

### The Main Lectin Receptor-Like Kinase Transcripts in B73 and B73-QTL Plants Are Fully Spliced

The B73 v4 and v5 genome annotations predicted two splice variants each of the L-RLK gene model on chromosome 10 (Supplementary Figure S3c and Supplementary Table S6). The splice variant that was common to both versions encoded the full-length 818 AA protein (B73 v5 L-RLK P2) that was similar to the 817 AA CML L-RLK. The alternative transcript in the version 4 annotation encoded a 698AA protein (AQK46433) (designated B73 v4 L-RLK P1). This predicted protein lacks the kinase active site (Supplementary Table S5) due to alternative splicing that removes exons 5 and 6 (Supplementary Figure S3c). This predicted transcript and protein have been removed by the annotators in B73 RefGen_v5. The alternative transcript in B73 RefGen_v5 retains intron 5, which results in a stop codon immediately after exon 5 to encode a 666 AA truncated protein Zm00001e041728_P001 (designated B73 v5 L-RLK P1).

**FIGURE 5:**
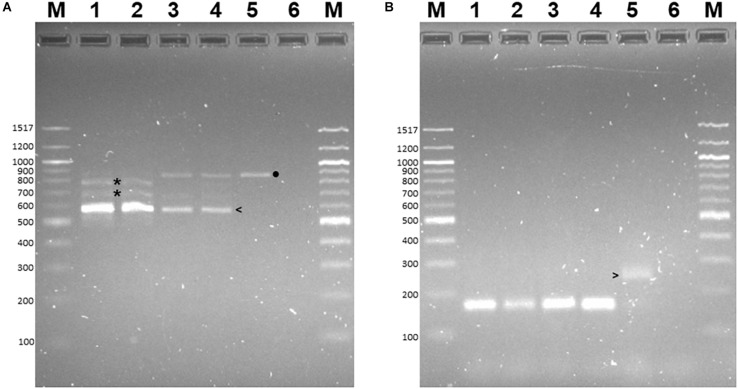
Reverse transcriptase-PCR of lectin receptor-like kinase (L-RLK) gene expression in B73 and B73-QTL maize plants inoculated with *C. zeina*. Maize leaves were either inoculated with *C. zeina* CMW25467 or mock-inoculated, and, at 43 dpi, samples were collected for RNA extraction and RT-PCR analysis. **(A)** Lanes 1–4 show RT-PCR of B73-QTL (mock), B73-QTL (*C. zeina*), B73 (mock), and B73 (*C. zeina*), respectively, with primers in exon 4 and exon 7 (L-RLK-qFor e4 and L-RLK-qRev e7 primers). Lane 5 template was B73 gDNA with the same primers. Lane 6 is a non-template control. Lanes M = 100 bp DNA Ladder (New England Biolabs), with sizes in bp shown to the left of each panel. **(B)** Lanes 1–4 show RT-PCR with control banana actin primers with the same templates in each lane as for Panel **(A)** to confirm that there was no gDNA in the cDNA samples. Lane 5 template was B73 gDNA with the actin primers. Lane 6 is a non-template control. Panel **(A)** shows that all four samples show the expected 593 bp product (left arrow) in which all three introns between exon 4 and exon 7 are spliced out, consistent with expression of the fully spliced transcript XM_008663415 (Zm00001e41728_T002 in B73 v5 genome). In addition, the B73-QTL samples have minor products of incomplete splicing in which either one intron is spliced out (776–794 bp products) or two introns are spliced out (676–694 bp products) (asterisks). The B73 samples (lanes 3 and 4) have a minor product of the same size as the 880 bp PCR product from gDNA (lane 5) (black dot). Panel **(B)** shows that all four samples (lanes 1–4) lack gDNA contamination since the expected 170 bp RT-PCR product is produced from the actin mRNA in which the intron has been spliced out. The gDNA PCR product is larger (260 bp) since it contains an intron (lane 5) (right arrow).

RT-PCR was conducted to determine if these alternative transcripts were expressed in B73 or B73-QTL plants when challenged with *C. zeina*. In this experiment, maize leaves were either mock-inoculated or inoculated with *C. zeina*, and then samples were collected at the same development stage of all plants at 43 dpi when mature GLS lesions were fully developed on the susceptible B73 plants. RT-PCR primers were designed in exon 4 and exon 7, and therefore they flanked introns 4, 5, and 6. The main RT-PCR product observed in all four samples was 593 bp, which corresponded to a transcript in which introns 4, 5, and 6 were spliced out, which would be expected from a full-length transcript of the L-RLK (Figure 5A, lanes 1–4). No RT-PCR product of 233 bp was observed in any sample, indicating that the splice variant encoding AQK46433 lacking the kinase site is not expressed under our conditions and supports the removal of this variant from the version 5 annotation of the B73 genome.

A minor amount of incomplete splicing was observed in B73-QTL plants (Figure 5A, lanes 1–2), producing RT-PCR products corresponding to retention of one of the three introns (676–694 bp products) or two of the three introns (776–794 bp products). One of the former products (693 bp), in which intron 5 is retained, corresponds to the transcript encoding the 666AA B73 v5 L-RLK P1. In B73 plants, the 880 bp RT-PCR minor product observed was the same size as the gDNA product (Figure 5A, lanes 3–5) indicating that none of the three introns was spliced out. We verified that this result was not due to gDNA in the RNA samples since a control RT-PCR with primers flanking an intron in the actin gene only produced a fully spliced product of 170 bp in all four samples (Figure 5B, lanes 1–4) and did not produce the 260 bp product shown in the PCR with B73 gDNA (Figure 5B, lane 5). Sanger sequencing confirmed that the 593 bp and 880 bp products lacked and contained the intron sequences, respectively (data not shown). The intermediate products were mixtures with either one or two introns retained (data not shown).

### The CML444 Copy of the Lectin Receptor-Like Kinase Gene Is Induced in B73-QTL Plants by *C. zeina* Challenge, but the B73 Copy Is Not

RT-qPCR analysis of glasshouse-grown maize was carried out to validate whether expression of the lectin receptor-like kinase gene was induced by *C. zeina* in B73-QTL plants and not induced in B73 plants, as indicated by the RNAseq data. In the field experiment, there was a developmental difference between lower leaves and upper leaves sampled, so this variable was eliminated in the glasshouse trial by collecting mock and *C. zeina*-inoculated leaves of the same age.

**FIGURE 6:**
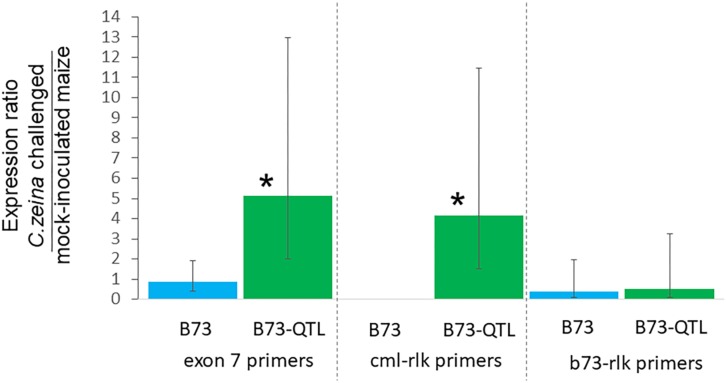
Reverse transcriptase-qPCR of lectin receptor-like kinase gene expression in B73 and B73-QTL maize plants inoculated with *C. zeina*. Maize leaves were either inoculated with *C. zeina* CMW25467 or mock-inoculated, and, at 43 dpi, samples were collected for RNA extraction and RT-qPCR analysis. The y-axis scale is the expression ratio of the maize lectin receptor-like kinase genes measured with the different primer pairs in maize leaves challenged with *C. zeina* compared to those mock-inoculated. Maize genotype treatments are labeled on the x-axis. The exon 7 primers (L-RLK e7 F1 and L-RLK e7 R1) amplify both the CML444 and the B73 copy of the gene. The cml-lrk primers (L-RLK e4e5 F1 [cml] and L-RLK e5 R1 [cml]) amplify the CML444 copy only, and thus there was no amplification in B73. The b73-lrk primers (L-RLK e4e5 F2 [b73] and L-RLK e5 R2 [b73]) amplify the B73 copy. Treatments where expression was significantly greater in the *C. zeina-*challenged samples than in the mock-inoculated samples based on a one-way ANOVA analysis with the Tukey-Kramer *post hoc* test (*p* < 0.05) are labeled with an asterisk above each column. The 95% confidence intervals are shown by error bars for each column.

RT-qPCR was carried out for the same samples as for the RT-PCR, with four biological replicates of each treatment. Expression was normalized to the *RPOL* gene that has previously been used for normalization of B73 maize gene expression in a GLS glasshouse trial (Christie et al., 2017). Lectin receptor-like kinase gene expression determined with the exon7 primers was five-fold higher in *C. zeina-*challenged B73-QTL leaves compared to mock-inoculated B73-QTL leaves at the same developmental stage, and the difference was statistically significant (*p* < 0.05; one way ANOVA, Tukey-Kramer test) (Figure 6). The expression values of individual treatments used to calculate these ratios are shown in Supplementary Figure S5A. This result corroborates the two-fold higher expression in *C. zeina-*challenged leaves compared to control leaves observed in the RNAseq field data (Table 2 and Supplementary Table S4). In contrast, the gene was expressed at a lower level in B73 plants and was not induced by *C. zeina* in the glasshouse experiment (Figure 6) (Supplementary Figure S5A). This is consistent with a previous RNAseq study of B73 field-grown plants that did not show differential expression of the gene between leaves with high and low *C. zeina* content (Supplementary Table S4) (Swart et al., 2017).

RT-qPCR with the CML gene copy-specific primers showed that the *cml-l-rlk* transcript was expressed four-fold higher in *C. zeina-*challenged compared to mock-inoculated leaves of B73-QTL (*p* < 0.05) (Figure 6) (Supplementary Figure S5B). No expression was detected in B73 plants, as expected from the CML-specific primers (Figure 6). The B73 gene copy-specific primers showed no significant difference in expression between *C. zeina-*challenged and mock-inoculated samples of either genotype (Figure 6 and Supplementary Figure S5C). Sanger sequencing of the RT-qPCR products confirmed that the exon 7 primers amplify the expected target sequence that is identical between the two sequences and that the other two primer pairs were gene copy-specific (data not shown).

### Synteny Analysis Reveals Duplication of Syntelogs of the Lectin Receptor-Like Kinase in Other Maize Lines

Synteny analysis was carried out, since one of the explanations for the two alleles of the lectin receptor kinase in CML444 (and B73-QTL) is adjacent gene duplication. This region of chromosome 10 shows synteny between five maize inbred lines, teosinte, and sorghum, with a BLH transcription factor and nuclear gene on one side and a protein kinase on the other (Supplementary Figure S6). Maize CML247, teosinte, and sorghum exhibit expansion in this region, with CML247 and sorghum showing additional lectin receptor-like kinase genes. In addition, full-length sequence alignments show greatest similarity between the CML444 allele and syntelogs from maize F7, teosinte, and sorghum (Supplementary Table S7).

BLASTP was used to identify the three most similar proteins in maize, other cereals, and *Arabidopsis thaliana* to the L-RLK-CML, and a phylogenetic tree was constructed using the maximum likelihood method (Supplementary Figure S7). This grouped L-RLK-CML in a clade with Zm00001d026382, as expected, together with the most similar maize paralog Zm00001d002174 (annotated as a cysteine-rich receptor-like protein kinase 6) and three proteins from *Setaria italica*. The three most similar Arabidopsis orthologs formed a separate clade.

## Discussion

The main outcome from this work was the development of a bioinformatics pipeline that can be used to identify novel expressed genes or alleles underlying a QTL using RNAseq data from near-isogenic lines with or without the QTL (Figure 1). This method is particularly effective if the background carrying the QTL introgression has a well-annotated genome sequence, such as the maize inbred line B73 (Jiao et al., 2017). For applications in molecular plant pathology, it is also helpful if the pathogen genome sequence is also available, as was the case for *C. zeina* (Wingfield et al., 2017). This allows mapping of RNAseq reads to both host and pathogen genomes simultaneously to identify unmapped reads that can be *de novo* assembled and annotated to find candidates from the QTL locus (Figure 1). In addition, differential expression analysis by mapping reads from biological replicates to a *de novo* “master assembly” of all reads in the experiment is used to determine whether the expression of candidate genes was correlated with higher levels of pathogen infection.

The main findings from our implementation of this pipeline to B73 plants introgressed with QTL10 for GLS disease resistance from inbred CML444 were: (i) B73-QTL was more resistant to GLS caused by the pathogen *C. zeina* in the field, as shown by AUDPC disease severity scores, *C. zeina* gDNA content, and GLS lesion area; (ii) *de novo* assembly of unmapped reads identified a candidate lectin receptor-like kinase named L-RLK-CML derived from the QTL10 region; (iii) expression of the lectin receptor-like kinase was correlated with *C. zeina* pathogen load and induced in B73-QTL plants but not in B73 lacking the QTL; (iv) several other B73-like or novel maize transcripts with a potential role in QDR conferred by QTL10 were identified.

Further investigation revealed that two copies of the lectin receptor-like kinase gene are present in CML444 and B73-QTL in contrast to there being a single gene in B73. The main copy corresponded to the *de novo* assembled transcript (*l-rlk-cml*), the candidate gene identified in this study. It encodes L-RLK-CML, an 817 AA lectin receptor-like kinase made up of a lectin ectodomain, a transmembrane domain, and an intracellular kinase domain with an active site signature (QIH29483). Gene-specific RT-qPCR showed that this copy was induced four-fold by *C. zeina* challenge. In contrast, the B73-like copy in B73-QTL was not induced by the pathogen. Similarly, in B73 plants, the single copy full-length transcript (Zm00001e041728_T002) encoding the full-length 818 AA protein (B73 v5 L-RLK P2) was expressed at a low level and not induced by *C. zeina* infection [an observation made from both field (RNAseq) and glasshouse (RT-qPCR) experiments].

Genome annotations of this lectin receptor-like kinase gene in B73 had computationally predicted splice variants. Our RT-PCR analysis showed that the major transcripts produced in both B73-QTL and B73 with or without fungal challenge corresponded to the fully spliced transcript that coded for the full-length proteins with all functional domains (817AA L-RLK-CML in B73-QTL and 818AA B73 v5 L-RLK P2 in B73). We found no evidence for the alternative splice variant (v4 annotation) encoding a 698AA protein B73 v4 L-RLK P1 (AQK46433) lacking the serine/threonine kinase active site signature. Our data are consistent with the fact that this splice variant was removed from v5 of the genome annotation. The only other splice variant in the v5 annotation had retention of intron 5, resulting in a stop codon immediately after exon 5 and a protein truncated by 152 AA at the C-terminus. Our RT-PCR analysis showed minor products corresponding to incomplete splicing of either one or two of the three introns between exon 4 and exon 7, which would include this splice variant. The biological significance of these products is questionable, as they encode non-functional proteins and are thus unlikely to play a positive role in disease resistance conferred by QTL10. A survey of splicing in B73 maize revealed that intron retention accounted for 56% of alternative splicing events (Mei et al., 2017). Intron retention was also the most abundant form observed in Arabidopsis RNAseq data, but many were associated with low numbers of reads, which may be due to incomplete splicing (Marquez et al., 2012). It is therefore possible that the intron retention variants observed in our study represent incompletely spliced pre-mRNA molecules captured in the RNA extraction process and not final products.

The candidate gene identified in this study encoding L-RLK-CML had 53 non-synonymous and 16 synonymous mutations compared with the B73 counterpart Zm00001e041728_T002 encoding B73 v5 L-RLK P2, which was annotated as a G-type lectin S-receptor-like serine/threonine-protein kinase. Domain analysis of L-RLK-CML showed that it contained all the functional domains of a G-type lectin receptor-like kinase (extracellular mannose-binding lectin, S-locus glycoprotein, and PAN-like domains, a trans-membrane domain, and the intracellular kinase domain). G-type receptor-like kinases (previously categorized as Bulb- or B-type due to their frequent occurrence in bulb species) are one of three classes of lectin receptor-like kinases (Teixeira et al., 2018). The best-characterized representatives of G-type L-RLKs are those that function in self-incompatibility, which is a mechanism that deters inbreeding in angiosperms (Kusaba et al., 2001). Self-incompatibility G-type L-RLKs in the genus Brassica were the first to be described with the S-locus glycoprotein domain. Therefore, in Arabidopsis, they are named S-Domain (SD1-X) proteins (Xing et al., 2013).

There are an estimated 38 G-type L-RLK SD1 genes in Arabidopsis (Teixeira et al., 2018), and therefore they do not only play a role in self-incompatibility. They are involved in a range of developmental and environmental stress responses, including response to biotic stress (Vaid et al., 2013). Therefore, there are precedents for G-type L-RLKs in disease resistance, as we are hypothesizing for L-RLK-CML in maize. The Arabidopsis LORE protein (SD1-29) was recently identified as a pattern-recognition receptor for lipopolysaccharide (LPS) from bacterial pathogens (Ranf et al., 2015). A second example in Arabidopsis is the RFO3 protein, which confers quantitative resistance to the fungus *Fusarium oxysporum f.sp. matthioli* (Cole and Diener, 2013). A third example is a G-type L-RLK in rice, Pi-d2, that confers resistance to the fungus *Magnaporthe grisea* (Chen et al., 2006). A fourth example is a G-type L-RLK in tobacco, Nt-Sd-RLK, the expression of which is induced by LPS (Sanabria et al., 2012).

Phylogenetic analysis indicated that the three closest Arabidopsis orthologs to L-RLK-CML were SD1-2, SD1-7, and At4G21390, although they formed a distinct clade from other cereal G-type L-RLKs. None of these are the self-incompatibility protein in Arabidopsis (which is SD1-8), but SD1-7 falls in the same clade and SD1-2 is in an adjacent clade to SD1-8 [according to Supplementary Figure S5a from Ranf et al. (2015)]. However, phylogenetic clustering does not appear to reflect similar functions due to neofunctionalization of paralogs. For example, AT4G21390 expression is induced in response to elicitors from *Botrytis* and *Phytophthora*, SD1-7 is upregulated by Fusarium, salt, and flg22, and SD1-2 is induced by cold (Chae et al., 2009).

We noted that B73-QTL plants had two copies of the L-RLK gene, the candidate allele encoding L-RLK CML and a B73-like gene. This is in contrast to B73, which only has one copy. One explanation is that B73-QTL is heterozygous for this locus; however, this is highly unlikely for several reasons. First, the parental line CML444, which is inbred (Messmer et al., 2009), also had the same pattern of two copies of the gene. Second, B73-QTL was developed by three cycles of backcross breeding and two cycles of selfing. Progeny selected at the last two cycles of selfing were homozygous for the two QTL flanking markers and the two internal markers, making it highly unlikely that the gene region was maintained in the heterozygous state. Finally, synteny analysis indicated that there is precedent for duplications of the gene, as shown for sorghum, teosinte, and maize CML247. It is not known whether CML247 is part of the pedigree of CML444, although the L-RLK-CML showed greater amino acid identity to the corresponding proteins of sorghum and teosinte compared to CML247.

The observation that the CML444 allele is most similar to teosinte and sorghum orthologs indicates that this allele might be older than the B73 allele. After duplication, it may have undergone subfunctionalization or neofunctionalization (Hughes et al., 2014), and one copy may have been lost in B73. Maize went through whole-genome duplications that were reduced back to diploidy, and the redundant genes have, in many cases, been adapted to new functions (Schnable et al., 2011; Hughes et al., 2014). For example, a maize QTL for resistance against corn ear-worm is caused by such a case (Zhang et al., 2003). Two genes originated from a duplication event act in combination to confer the resistance (Zhang et al., 2003). This might also be occurring in the QTL10 region. It could be that instead of the CML444 allele being more effective than the B73 allele, they improve each other’s functionality when defending against *C. zeina* infection.

Although the L-RLK-CML is the strongest candidate for conferring the major effect of QTL10 against *C. zeina*, it is possible that the QDR may depend on another of the candidates identified in this study. Even though their closest B73 match is not positioned within the QTL region, these candidates may actually be within the QTL in CML444 due to differences in genomic organization compared to B73 (Albert et al., 2019). It is also feasible that the QTL is made up of a combination of tightly linked genes (Nelson et al., 2018).

The other candidate genes have functions that are consistent with quantitative resistance against a foliar pathogen such as *C. zeina*. They include functions that have been found in QDR against other maize pathogens, such as a wall-associated kinase, which confers resistance to head smut in maize (Zuo et al., 2014). GLS disease is characterized by necrosis during lesion development, so it is thought that toxin production is an important aspect of pathogenicity. Detoxification enzymes such as GSTs and flavin mono-oxygenase have been associated with resistance to GLS (Wisser et al., 2011; Benson et al., 2015), and the identification of two GSTs from the *de novo* assemblies is consistent with this. It is intriguing that our study reported a remorin that was down-regulated in response to *C. zeina*, since another study reported a remorin underlying QDR to northern corn leaf blight in maize (Jamann et al., 2016).

Callose deposition is a well-known plant protection mechanism against fungi, and in a previous study of maize co-expression networks in response to *C. zeina*, a callose synthase was the most highly induced transcript in a co-expression module associated with QDR (Christie et al., 2017). In the current study, a callose-binding plasmodesmata protein was one of the candidates. In the previous study, calcium signaling was identified in the transcriptome response to *C. zeina*. In this study, a calcineurin-B-like protein, two calmodulins, and a calcium-dependent lipid-binding protein were expressed in B73-QTL in response to the pathogen. Vesicular trafficking is regarded as an important part of plant defense so that anti-microbial cargoes can be delivered to sites of infection, including small RNAs; therefore, it is significant that a tetraspanin and an exocyst component SEC6 were identified (Jimenez-Jimenez et al., 2019).

Recently, a G-type L-RLK in Arabidopsis SD1-13 (At1g11330) was implicated in immune responses and suppression of ABA signaling (Park et al., 2019). The link between ABA signaling and resistance to *C. zeina* is relevant since the fungus penetrates maize leaves through the stomata, and therefore regulation of stomatal opening is a possible mechanism of defense against this pathogen. One of the enriched GO terms in B73-QTL was “stomatal closure.” It is therefore intriguing that two abscisic stress ripening proteins were included in the *de novo* assembled transcripts. Finally, several other receptor or signal transduction candidates were identified including two LRR receptor kinases (NBS-LRRs). Ethylene is an important defense hormone, especially against necrotrophic pathogens, and therefore it is notable that a key enzyme in ethylene biosynthesis 1-aminocyclopropane-1-carboxylate oxidase was included (Houben and Van de Poel, 2019). PR proteins are important components of fungal defense, and our study identified three candidates – a glucan beta-glucosidase, a chitinase and a lipid transfer protein, consistent with a previous study (Christie et al., 2017).

The limitations of our study, which fall outside of the scope of this work, as it focuses on bioinformatics applications in molecular plant pathology, are the provision of further biological experimental evidence of the candidate genes, especially the lectin receptor-like kinase. The methods to be followed to obtain this evidence would be (i) further fine-mapping of the QTL region to reduce the potential number of candidate genes, and (ii) knockout of candidate genes. Unfortunately, the maize mutant library resources, such as UniformMu in the W22 background, do not currently have a mutant in the gene model Zm00001d026382 (Settles et al., 2007). Another approach would be virus-induced gene silencing using the Foxtail mosaic virus (FoMV) vector (Mei et al., 2016). In test experiments, CML444 was not susceptible to FoMV (Berger, unpublished). Transformation of maize with a silencing construct or gene editing could be deployed; however, maize transformation is highly genotype-specific, and a tissue culture and transformation protocol has not yet been developed for CML444.

In our study, we applied three filtering steps to identify candidate genes, namely (i) position within the QTL region, (ii) plant immunity-related annotation, and (iii) induction by the pathogen. However, it should be borne in mind that the latter two criteria should be applied with care. For example, a QTL causal gene could have a novel function (Poland et al., 2009). However, a count of the 30 QDR causal genes listed in the Supplementary Material of Nelson et al. (2018) showed that most of them (18) had a plant defense-related annotation. It is also possible that a causal gene is not induced by the pathogen. Many plant-resistance genes are constitutively expressed but subject to post-translational regulation (Withers and Dong, 2017). However, in support of our approach, there are several examples of pathogen-induced or spatio-temporally regulated expression of QDR causal genes, such as maize *ZmWAK*, wheat *Yr36*, genes at the soybean *rhg1* locus, and rice *RKS1* (French et al., 2016).

Finally, our study has provided a bioinformatics pipeline for identification of candidate genes underlying a QTL and has revealed the lectin receptor-like kinase as the strongest candidate, with additional alternative candidates. Lectin receptor-like kinases are currently a hot topic in plant immunity (Van Holle and Van Damme, 2018). Moreover, the bioinformatics strategy developed in this work is not only specific to the identification of QDR candidates but can be applied to any trait QTL in any plant species, provided that near-isogenic lines with and without the QTL are available, and thus should have broad applicability.

## Data Availability Statement

The datasets generated for this study can be found in the NCBI GEO (Acc. GSE137198), NCBI (Acc. MT108451 and QIH29483) Zenodo, Supplementary Material.

## Author Contributions

DB conceived the study. DB, YV, and RP participated in the design and management. YV and LS provided the resources. TW and LS performed the bioinformatics analyses. TW, VS, and KS conducted the experiments. TW and DB interpreted the data and wrote the manuscript. All authors have read and approved the final manuscript.

## Conflict of Interest

The authors declare that the research was conducted in the absence of any commercial or financial relationships that could be construed as a potential conflict of interest.

## Footnotes

1 https://fairsharing.org/biodbcore-001265/

2 www.maizegdb.org

3 www.qbaseplus.com
