# Supplementary material for: De novo Assembly of Transcriptomes From a B73 Maize Line Introgressed With a QTL for Resistance to Gray Leaf Spot Disease Reveals a Candidate Allele of a Lectin Receptor-Like Kinase
Source: Front Plant Sci. 2020 Mar 13;11:191. doi: 10.3389/fpls.2020.00191 (PMC7083176; doi:10.3389/fpls.2020.00191)
Supplement: Supplementary file 1 [file Data_Sheet_1.pdf]

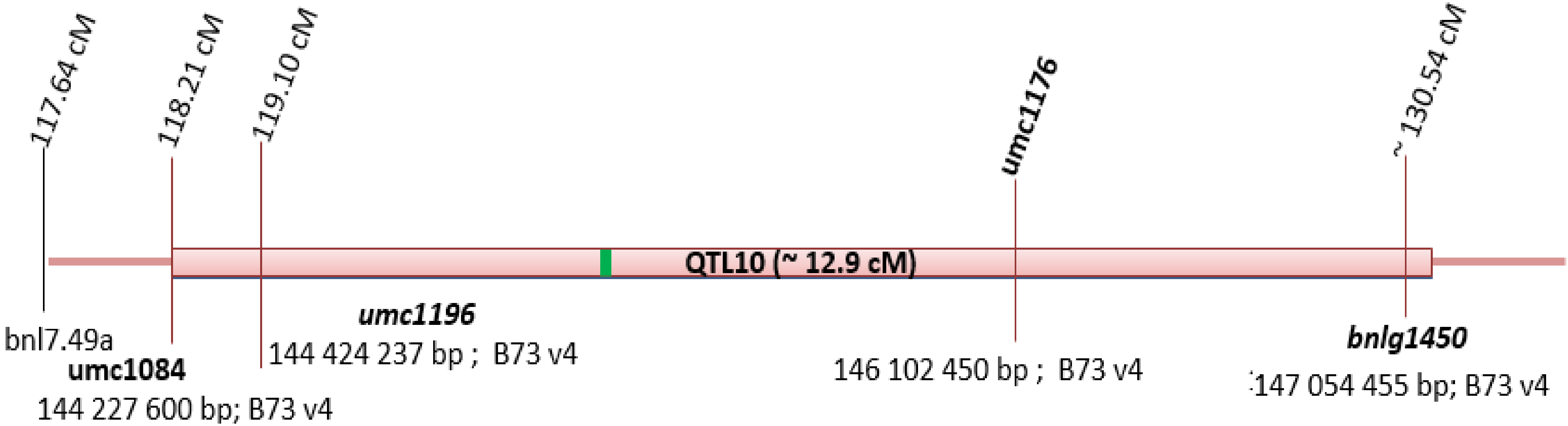

Figure S1. Physical map of a chromosome 10 region in maize line B73 corresponding to QTL10 from CML444. The B73 physical map (v4) positions of four SSR markers used for marker assisted backcross breeding to introgress the region from CML444 into B73 to produce B7-QTL are shown. The genetic map size of the QTL10 region in cM is indicated (Berger et al. 2014).

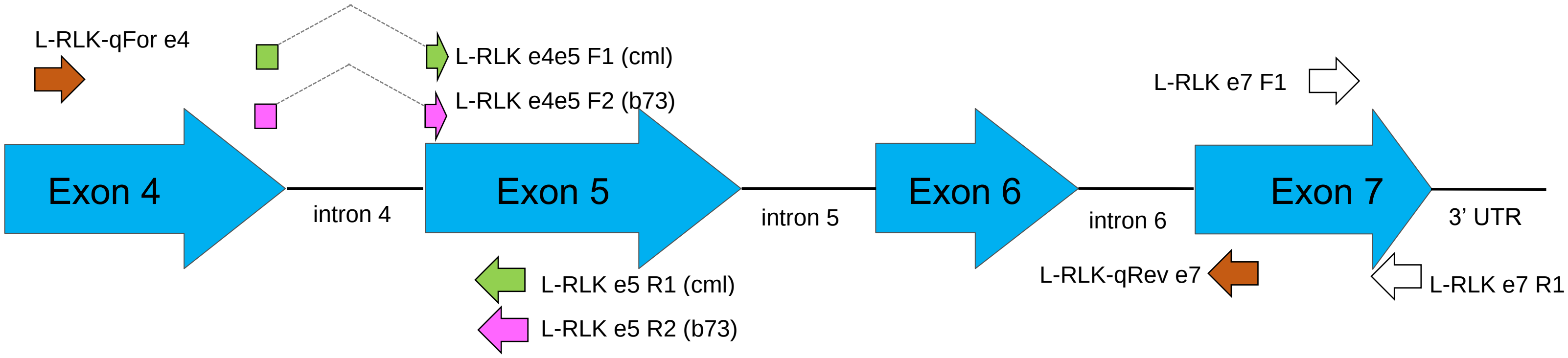

Figure S2a. Primer positions relative to the maize B73 L-RLK gDNA exon4-exon7 region. The primers L-RLK-qFor e4 and L-RLK-qRev e7 were used in RT-PCR to determine splice variants. The primers L-RLK e7 F1 and L-RLK e7 R1 were used for expression analysis by RT-qPCR. L-RLK e4e5 F1 (cml) and L-RLK e5 R1 (cml) were used for RT-qPCR expression analysis of the CML444 copy of the L-RLK gene. L-RLK e4e5 F2 (b73) and L-RLK e5 R2 (b73) were used for RT-qPCR expression analysis of the B73 copy of the L-RLK gene. Sequences are shown in Supplementary Figure S2b. The gDNA sequence shown (1427 bp) is identical in both versions of the B73 genome (Zm00001d026382 [v4]; Zm00001e041728 [v5]).

```
                          1  Exon 4                                L-RLK-qFor e4 primer  5' TGCCGAAGAGTTCAGGAATG 3'             99
CML L-RLK cDNA      (1)  GAATGTTGGAAGGAACCAAAGAAGTAGCCATCAAGAGACTGAGCAAGAATTCTGGGCAAGGTGCCGAAGAGTTCAGGAATGAAGTAGTTTGATTGCAA
B73 L-RLK T2        (1)  GAATGTTGGAAGGAACCAAAGAAGTAGCCATCAAGAGACTGAGCAAGAGTTCTGGGCAAGGTGCCGAAGAGTTCAGGAATGAAGTAATCTTGATTGCAA
B73 L-RLK gDNA      (1)  GAATGTTGGAAGGAACCAAAGAAGTAGCCATCAAGAGACTGAGCAAGAGTTCTGGGCAAGGTGCCGAAGAGTTCAGGAATGAAGTAATCTTGATTGCAA
                                             100                                                                              198
CML L-RLK cDNA    (100)  AGTTGCAGCACAAGAACCTAGTTAAGCTTCTTGGTTTCTGTGTTCATGAAGATGAGAAGATGTTAGTTTATGAGTACTTGCCGAACAAAAGCTTAGATT
B73 L-RLK T2      (100)  AGTTGCAGCACAAGAACCTAGTTAAGCTTCTTGGTTGCTGTGTTCATGAAGATGAGAAGCTGTTAGTTTATGAGTACTTGCCGAACAAAAGCTTAGATT
B73 L-RLK gDNA    (100)  AGTTGCAGCACAAGAACCTAGTTAAGCTTCTTGGTTGCTGTGTTCATGAAGATGAGAAGCTGTTAGTTTATGAGTACTTGCCGAACAAAAGCTTAGATT

                         199                                                                                              297
                        5'CTTCCTCTTTGACTCTTC 3'   L-RLK e4e5 F1 (cml) primer
CML L-RLK cDNA    (199)  ACTTCCTCTTTG---------------------------------------------------------------------------------------

                        5'CTTCCTCTTTGATTCTGC 3'   L-RLK e4e5 F2 (b73) primer
B73 L-RLK T2      (199)  ACTTCCTCTTTG--------------------------------Intron 4-----------------------------------------------
B73 L-RLK gDNA    (199)  ACTTCCTCTTTGGTACATTGAGTTTTAAAACTTTCCATTTAGTTAACAAAATCAACAAATATACATGCATATGCCTTCTGATATGTATGCCTTGTTTCA


                         298           Exon 5                                                                             396
                           5'CTTCCTCTTTGACTCTTC 3' L-RLK e4e5 F1 (cml) primer
CML L-RLK cDNA    (211)  ---------------ACTCTTCAAGAAAGTCAACGCTTCAGTGGCCAACAAGGTTCAAGATAATCCACGGGGTAGCTAGAGGAATTATGTATCTTCATC

                           5'CTTCCTCTTTGATTCTGC 3' L-RLK e4e5 F2 (b73) primer
B73 L-RLK T2      (211)  ---------------ATTCTGCAAGAAAGTCAACGCTTCAGTGGCCAACAAGGTTCAAGATAATCCACGGGGTAGCTAGAGGAATTATGTATCTTCATC
B73 L-RLK gDNA    (298)  TTTTATTCATATTAGATTCTGCAAGAAAGTCAACGCTTCAGTGGCCAACAAGGTTCAAGATAATCCACGGGGTAGCTAGAGGAATTATGTATCTTCATC
```

```
                          397                                                                                               495
CML L-RLK cDNA   (295) AGGACTCGAGATTAACAATAATCCATAGAGATCTCAAAGCAAGCAACATCTTGTTAGATAAGGACATGAGCCCAAAAATATCAGATTTTGGCATGGCTA
                     3' GTCCTGAGCTCTAATTG 5' L-RLK e4e5 R1 (cml) primer

B73 L-RLK T2     (295) AAGACTCGAGATTAACAATAATCCATAGGGATCTCAAAGCAAGCAACATCTTGTTAGATAAGGACATGAGCCCAAAAATATCAGATTTTGGCATGGCTA
                     3' GTTCTGAGCTCTAATTG 5' L-RLK e4e5 R2 (b73) primer
B73 L-RLK gDNA   (397) AAGACTCGAGATTAACAATAATCCATAGGGATCTCAAAGCAAGCAACATCTTGTTAGATAAGGACATGAGCCCAAAAATATCAGATTTTGGCATGGCTA

                          496                                                                                               594
CML L-RLK cDNA   (394) GAATATTCAGTAGCGACCAGCTCCATGCAAATACTAACCGAGTTGTAGGGACATA--------------------------------------------
B73 L-RLK T2     (394) GAATATTCAGTAGCGACCAGCTCCATGCAAATACTAACCGAGTTGTAGGCACATA-----Intron 5-------------------------------
B73 L-RLK gDNA   (496) GAATATTCAGTAGCGACCAGCTCCATGCAAATACTAACCGAGTTGTAGGCACATAGTAAGTAACACCGGCATTGCCAACTCTTCATATATATTGAACTA

                          595                                                        Exon 6                                 693
CML L-RLK cDNA   (449) ---------------------------------------------------------TGGTTACATGTCTCCTGAATATGCAATGGAAGGTGCCTTTTC
B73 L-RLK T2     (449) ---------------------------------------------------------TGGTTACATGTCTCCTGAATATGCAATGGAAGGTGCCTTTTC
B73 L-RLK gDNA   (595) ATTTTGTTTGAAGTTTCACATATATGCAAACTTTTTTTTGTGAAATCATTTGCACAGTGGTTACATGTCTCCTGAATATGCAATGGAAGGTGCCTTTTC

                          694                                                                                               792
CML L-RLK cDNA   (491) AGTCAAGTCTGACACTTACAGCTTTGGTGTTTTAATGCTGGAGATTGTAAGTGGTTTGAAGATCAGCTCACCACATCTTCACATGGATTTTCCTAATCT
B73 L-RLK T2     (491) AGTCAAGTCTGACACTTACAGCTTTGGTGTTTTAATGCTGGAGATTGTAAGTGGTTTGAAGATCAGCTCACCACATCTTCACATGGATTTTCCTAATCT
B73 L-RLK gDNA   (694) AGTCAAGTCTGACACTTACAGCTTTGGTGTTTTAATGCTGGAGATTGTAAGTGGTTTGAAGATCAGCTCACCACATCTTCACATGGATTTTCCTAATCT

                                                                                                                         Exon 7
                          793                                                                                               891
CML L-RLK cDNA   (590) TAGAGCATATG------------------------------------------------------------------------------------CATG
B73 L-RLK T2     (590) TAGAGCATATG------Intron 6--------------------------------------------------------------------CATG
B73 L-RLK gDNA   (793) TAGAGCATATGTAAGGACTGGCACACTCTCATAATAATTCAGAACTGTCCACATGATAAAAACGATCCTGATATGAGATTGTGTCATGTTTCAGGCATG

                          892                                                                                               990
CML L-RLK cDNA   (605) GAACATGTGGAAAGAAGGAAAAATAGAAGATTTGGTGGACTCATCAGTCATGGAGAATTGCTCCCTTGATGAAGTTTCACAATGTGTCCATATAGGACT
B73 L-RLK T2     (605) GAACATGTGGAAAGAAGGGAAAATAGAAGATTTGGTGGACTCATCAGTCATGGAGAATTGCTCCCTTGATGAAGTTTCACAATGTGTACATATAGGACT
B73 L-RLK gDNA   (892) GAACATGTGGAAAGAAGGGAAAATAGAAGATTTGGTGGACTCATCAGTCATGGAGAATTGCTCCCTTGATGAAGTTTCACAATGTGTACATATAGGACT
                          L-RLK-qFor e4 primer  3’ CTAAACCACCTGAGTAGTCAGT 5’


                          991                                                    L-RLK e7 F1 5‘ AACGACGACACTTCCAACTC 3‘   1089
CML L-RLK cDNA   (704) CTTGTGTGTTCAAGACAGTCCGAGCTTCAGGCCACTCATGTCGGCGGTTGTGTCCATGCTGGAGAACAAAACGACAACACTTCCAACTCCAAGTCAACC
B73 L-RLK T2     (704) CTTGTGTGTTCAAGACAGTCCGAGCTTCAGGCCACTCATGTCGGCGGTTGTGTCCATGCTGGAGAACAAAACGACGACACTTCCAACTCCAAGTCAACC
B73 L-RLK gDNA   (991) CTTGTGTGTTCAAGACAGTCCGAGCTTCAGGCCACTCATGTCGGCGGTTGTGTCCATGCTGGAGAACAAAACGACGACACTTCCAACTCCAAGTCAACC

                          1090                                                                                              1188
CML L-RLK cDNA   (803) TGTATATTTTGCAGTTAGGGACCCCTACCAACCTGGAAAGGCAGTTGGCAACAAGGAGTTGTCTATATATGATATGAGTCTCACTGTGCCAGAAGGTCG
B73 L-RLK T2     (803) TGTATATTTTGCAGTTAGGGACCCCTACCAACCTGGAAAGGCAGTTGGCAACAAGGAGTTGTCTATATATGATATGAGTCTCACTGTGCCAGAAGGTCG
B73 L-RLK gDNA  (1090) TGTATATTTTGCAGTTAGGGACCCCTACCAACCTGGAAAGGCAGTTGGCAACAAGGAGTTGTCTATATATGATATGAGTCTCACTGTGCCAGAAGGTCG
                                                                                   L-RLK e7 R1 3‘ CTATACTCAGAGTGACACGGTC 5‘
```

```
                          1189                                                                                                1287
CML L-RLK cDNA    (902)  TTAAATGCTTTATTTTTATAACTGATTGGGAGGATTAATTAAACCCCACTGGCATATTCATTGCATAACTAAAACTGATCGTTCAGCCCATGTGAAGGT
B73 L-RLK T2      (902)  TTAAATGCTTTATTTTTATAACTGATTGGGATGATTAATTAAACCCCACTGGCATATTCATTGCATAACTAAAACTGATCGTTCAGCCCATGTGAAGGT
B73 L-RLK gDNA   (1189)  TTAAATGCTTTATTTTTATAACTGATTGGGATGATTAATTAAACCCCACTGGCATATTCATTGCATAACTAAAACTGATCGTTCAGCCCATGTGAAGGT

                         1288                                                                                                 1386
CML L-RLK cDNA   (1001)  TATGTGAATGGTCGGTAATTTGCATTTTGCAACAGTTTGGTAAGCCTGCTTATATAATTGTATAAAGACAGGTGATTTCCTTCTCTCAGCTCATGTGGT
B73 L-RLK T2     (1001)  TATGTGAATGGTCGGTAATTTGCATTTTGCAACAGTTTGGTAAGCCTGCTTATATAATTGTATAAAGACAGGTGATTTCCTTCTCTCA-----------
B73 L-RLK gDNA   (1288)  TATGTGAATGGTCGGTAATTTGCATTTTGCAACAGTTTGGTAAGCCTGCTTATATAATTGTATAAAGACAGGTGATTTCCTTCTCTCAGCTCATGTGGT

                          1387                                    1427
CML L-RLK cDNA   (1100)  TGTACTGTTTATGTGCTTATTATGTGGATCCAATTTGACCG
B73 L-RLK T2     (1089)  -----------------------------------------
B73 L-RLK gDNA   (1387)  TGTACTGTTTATGTGCTTATTATGTGGATCCAATTTGACCG
```

Figure S2b. Nucleotide alignments to illustrate maize L-RLK gene-specific primer design. The maize B73 L-RLK gDNA exon 4–exon 7 region (B73 L-RLK gDNA) is aligned with corresponding regions of the fully spliced B73 L-RLK T2 transcript (XM_008663415, Zm00001e041728_T002) and the CML L-RLK cDNA (de novo assembled transcript in this study; MT108451). Introns are labeled in yellow. Nucleotides in B73 that are different from CML444 are marked blue. SNPs between B73 and CML at the start of exon 5 were used to design gene copy-specific exon-exon primers with differences in their 3' ends [L-RLK e4e5 F1 (cml) and L-RLK e4e5 F2 (b73)]. There was also one nucleotide difference in the 3' end of the reverse primers [L-RLK e5 R1 (cml) and L-RLK e5 R2 (b73)]. The primers specific to the CML copy are marked in green, and the primers specific to the B73 copy are marked in purple. The positions of the RT-PCR primers (L-RLK-qFor e4 and L-RLK-qRev e7) to distinguish splice variants and the RT-qPCR primers for expression analysis (L-RLK e7 F1 and L-RLK e7 R1) are also shown. The stop codon is marked in red.

```
                                   1                                                                                                  100
CML444 L-RLK-CML (817aa)    (1)   MMYVPIFILLVLLSSLCQSDDQLTHTKPLSPKDTLISAGGDFALGFFSPTSSSNKLYIGIWYNNVPERERTVVWIANRDSPVTTPATSVKLAVTNDSELV
B73 v5 L-RLK P2 (818aa)     (1)   MMYVPIFILLVVLSSLCQSDDQLTHTKPLFPKETLISAGGDFALGFFSPTSSSNKLYIGIWYNNVPERERTVVWIANRDSPVTTPATSVKLAVTNDSELV
B73 v5 L-RLK P1 (666aa)     (1)   MMYVPIFILLVVLSSLCQSDDQLTHTKPLFPKETLISAGGDFALGFFSPTSSSNKLYIGIWYNNVPERERTVVWIANRDSPVTTPATSVKLAVTNDSELV
B73 v4 L-RLK P1 (698aa)     (1)   MMYVPIFILLVVLSSLCQSDDQLTHTKPLFPKETLISAGGDFALGFFSPTSSSNKLYIGIWYNNVPERERTVVWIANRDSPVTTPATSVKLAVTNDSELV
                                   101                                                                                                200
CML444 L-RLK-CML (817aa)  (101)   LSDSQGNIFWMATSSTSGGAGAFAVLLNSGNFVLRSPNGTDVWQSFDHPTDTLLPTMRFGISYRSQPATRLFAWKGPDDPSTGNISFSLDPASSNLQMFI
B73 v5 L-RLK P2 (818aa)   (101)   LSDSQGHIFWMATSSTSGGAGAFAVLLNSGNFVLRSPNGTDVWQSFDHPTDTVLPTMRSMIRYRSQPATGLFAWKSPDDPSTGDISSGMDPASSNVQVFI
B73 v5 L-RLK P1 (666aa)   (101)   LSDSQGHIFWMATSSTSGGAGAFAVLLNSGNFVLRSPNGTDVWQSFDHPTDTVLPTMRSMIRYRSQPATGLFAWKSPDDPSTGDISSGMDPASSNVQVFI
B73 v4 L-RLK P1 (698aa)   (101)   LSDSQGHIFWMATSSTSGGAGAFAVLLNSGNFVLRSPNGTDVWQSFDHPTDTVLPTMRSMIRYRSQPATGLFAWKSPDDPSTGDISSGMDPASSNVQVFI
                                   201                                                                                                300
CML444 L-RLK-CML (817aa)  (201)   WNGTLPYFRSSVVNDVSVSGGTYQANNTSVTYQGILVDTGDEIYYTFTVLAGSPYMHFSLHYTGKMRILAWENSTSSWVVIFEGPSVGCDLYASCGPFGY
B73 v5 L-RLK P2 (818aa)   (201)   WNGTLPYYRSSFFNDVPASGGTYQANNTSVTYQWMMVDTGDEIYYTFTVLAGSPYMHFSLHHTGKMRLLVWENSTSSWVLISEEPSVGCDLYASCGPFGY
B73 v5 L-RLK P1 (666aa)   (201)   WNGTLPYYRSSFFNDVPASGGTYQANNTSVTYQWMMVDTGDEIYYTFTVLAGSPYMHFSLHHTGKMRLLVWENSTSSWVLISEEPSVGCDLYASCGPFGY
B73 v4 L-RLK P1 (698aa)   (201)   WNGTLPYYRSSFFNDVPASGGTYQANNTSVTYQWMMVDTGDEIYYTFTVLAGSPYMHFSLHHTGKMRLLVWENSTSSWVLISEEPSVGCDLYASCGPFGY
                                   301                                                                                                400
CML444 L-RLK-CML (817aa)  (301)   CDLTIAMPTCQCPDHFELVDSLNFSRGCQRKEELKCGTDSYFMTMPSMKIPDKFLHIRNRTFDQCAAECARNCTCTAYAYANLSTAGATGDTSRCLVWIQ
B73 v5 L-RLK P2 (818aa)   (301)   CDLTKAMPTCQCPDHFELVDSLNFSRGCQRKEELKCGTESYFMTMPNMKIPDKFLHIRNRTFDQCAAECARNCTCMAYTYATLSTASTTGDASRCLVWTQ
B73 v5 L-RLK P1 (666aa)   (301)   CDLTKAMPTCQCPDHFELVDSLNFSRGCQRKEELKCGTESYFMTMPNMKIPDKFLHIRNRTFDQCAAECARNCTCMAYTYATLSTASTTGDASRCLVWTQ
B73 v4 L-RLK P1 (698aa)   (301)   CDLTKAMPTCQCPDHFELVDSLNFSRGCQRKEELKCGTESYFMTMPNMKIPDKFLHIRNRTFDQCAAECARNCTCMAYTYATLSTASTTGDASRCLVWTQ
                                   401                                                                                                500
CML444 L-RLK-CML (817aa)  (401)   DLIDMEKASHLLENLYIRLGESPDQKS-NFQKILLSTIACLLLLTIGALGWACKIRGKWHKKKIQTRRMLEYLSSTDEAGGKNIEFSFIAFENIVTATDN
B73 v5 L-RLK P2 (818aa)   (401)   DLIDMEKASVFLENLYIRLGESPDQKKRNFQKILLSAIACLLLLTIGALGWACKIRGKWHKKKIQTRMMLEYLSSTDEAGGKNIEFPFITFENIVTATDN
B73 v5 L-RLK P1 (666aa)   (401)   DLIDMEKASVFLENLYIRLGESPDQKKRNFQKILLSAIACLLLLTIGALGWACKIRGKWHKKKIQTRMMLEYLSSTDEAGGKNIEFPFITFENIVTATDN
B73 v4 L-RLK P1 (698aa)   (401)   DLIDMEKASVFLENLYIRLGESPDQKKRNFQKILLSAIACLLLLTIGALGWACKIRGKWHKKKIQTRMMLEYLSSTDEAGGKNIEFPFITFENIVTATDN
                                   501                                                                                                600
CML444 L-RLK-CML (817aa)  (500)   FSDCNILGKGGFGKVYKGMLEGTKEVAIKRLSKNSGQGAEEFRNEVVLIAKLQHKNLVKLLGFCVHEDEKMLVYEYLPNKSLDYFLFDSSRKSTLQWPTR
B73 v5 L-RLK P2 (818aa)   (501)   FSDCNILGKGGFGKVYKGMLEGTKEVAIKRLSKSSGQGAEEFRNEVILIAKLQHKNLVKLLGCCVHEDEKLLVYEYLPNKSLDYFLFDSARKSTLQWPTR
B73 v5 L-RLK P1 (666aa)   (501)   FSDCNILGKGGFGKVYKGMLEGTKEVAIKRLSKSSGQGAEEFRNEVILIAKLQHKNLVKLLGCCVHEDEKLLVYEYLPNKSLDYFLFDSARKSTLQWPTR
B73 v4 L-RLK P1 (698aa)   (501)   FSDCNILGKGGFGKVYKGMLEGTKEVAIKRLSKSSGQGAEEFRNEVILIAKLQHKNLVKLLGCCVHEDEKLLVYEYLPNKSLDYFLFGT---------LS
                                   601                 Kinase active site                                                             700
CML444 L-RLK-CML (817aa)  (600)   FKIIHGVARGIMYLHQDSRLTIIHRDLKASNILLDKDMSPKISDFGMARIFSSDQLHANTNRVVGTYGYMSPEYAMEGAFSVKSDTYSFGVLMLEIVSGL
B73 v5 L-RLK P2 (818aa)   (601)   FKIIHGVARGIMYLHQDSRLTIIHRDLKASNILLDKDMSPKISDFGMARIFSSDQLHANTNRVVGTYGYMSPEYAMEGAFSVKSDTYSFGVLMLEIVSGL
B73 v5 L-RLK P1 (666aa)   (601)   FKIIHGVARGIMYLHQDSRLTIIHRDLKASNILLDKDMSPKISDFGMARIFSSDQLHANTNRVVGT*---------------------------------
B73 v4 L-RLK P1 (698aa)   (592)   FKTFH-----------------------------------------------------------------------------------------------
                                   701                                                                                                800
CML444 L-RLK-CML (817aa)  (700)   KISSPHLHMDFPNLRAYAWNMWKEGKIEDLVDSSVMENCSLDEVSQCVHIGLLCVQDSPSFRPLMSAVVSMLENKTTTLPTPSQPVYFAVRDPYQPGKAV
B73 v5 L-RLK P2 (818aa)   (701)   KISSPHLHMDFPNLRAYAWNMWKEGKIEDLVDSSVMENCSLDEVSQCVHIGLLCVQDSPSFRPLMSAVVSMLENKTTTLPTPSQPVYFAVRDPYQPGKAV
B73 v5 L-RLK P1 (666aa)   (667)   ----------------------------------------------------------------------------------------------------
B73 v4 L-RLK P1 (698aa)   (597)   ----------------LAWNMWKEGKIEDLVDSSVMENCSLDEVSQCVHIGLLCVQDSPSFRPLMSAVVSMLENKTTTLPTPSQPVYFAVRDPYQPGKAV
                                   801             818
CML444 L-RLK-CML (817aa)  (800)   GNKELSIYDMSLTVPEGR*
B73 v5 L-RLK P2 (818aa)   (801)   GNKELSIYDMSLTVPEGR*
B73 v5 L-RLK P1 (666aa)   (667)   ------------------
B73 v4 L-RLK P1 (698aa)   (681)   GNKELSIYDMSLTVPEGR*
```

Figure S3a. Amino acid alignment of predicted G-lectin S-receptor-like kinase proteins from maize B73 [B73 v5 L-RLK P2 (818aa) annotated in both the v4 genome (XP_008661637) and v5 genome (Zm00001e041728_P001), B73 v5 L-RLK P1 (666aa) (Zm00001e041728_P001), B73 v4 L-RLK P1 (698aa)(AQK46433)], and the protein predicted from the de novo assembled transcript (MT108451) from B73-QTL (CML444 L-RLK-CML; QIH29483).

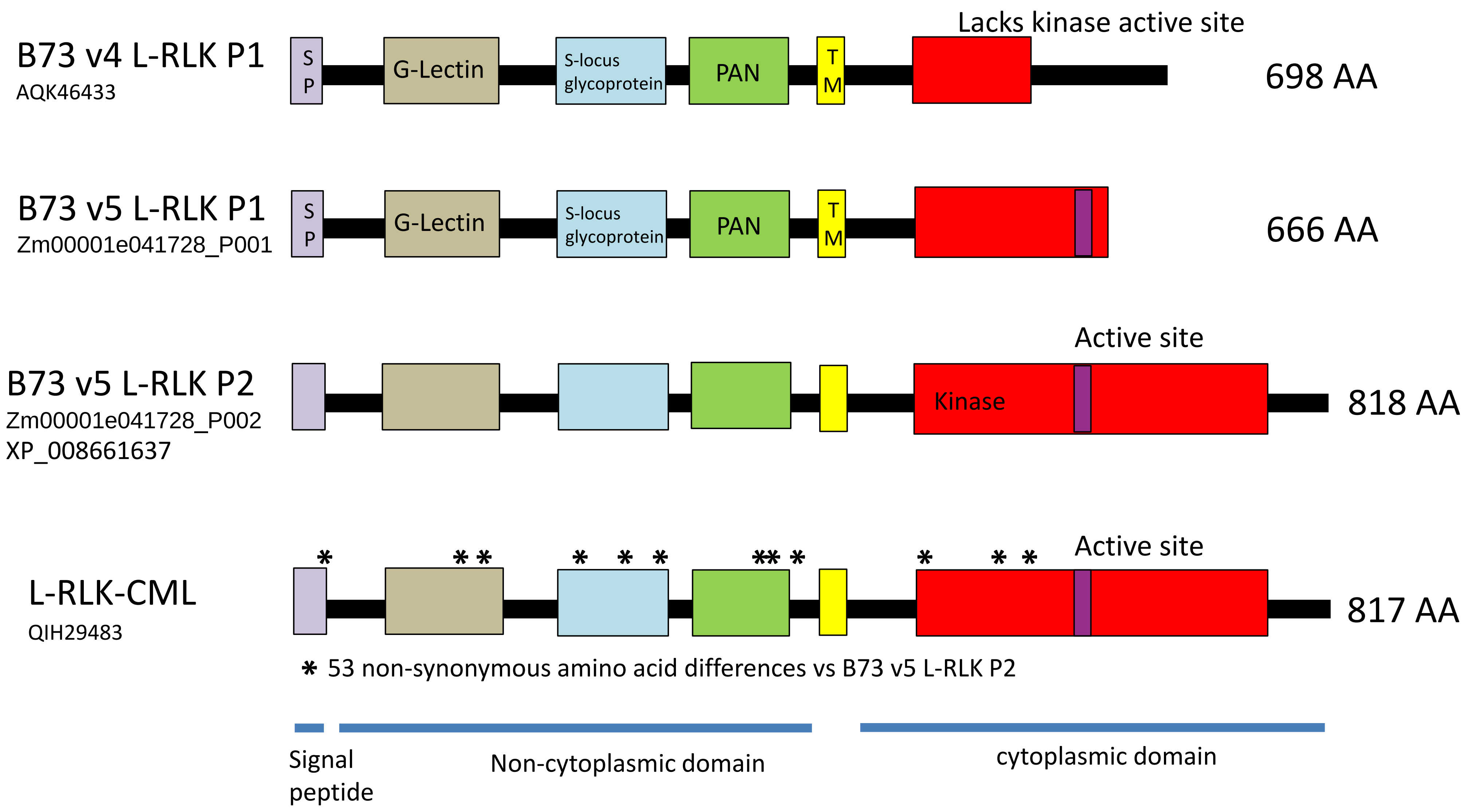

Figure S3b. Domain structures of predicted G-lectin S-receptor-like kinases from maize B73 genome annotations: B73 v4 L-RLK P1 (AQK46433), B73 v5 L-RLK P1 (Zm00001e041728_P001), B73 v5 L-RLK P2 (Zm00001e041728_P002, XP_008661637), and the de novo assembled transcript (MT108451) from B73-QTL (L-RLK-CML; QIH29483). Domains: SP = Signal peptide (purple); G-lectin (brown); S-locus glycoprotein (blue), PAN-like domain (green), trans-membrane (TM), kinase (red); kinase active site (dark purple). Drawn to scale. AQK464433 was removed from the v5 annotation of the B73 genome.

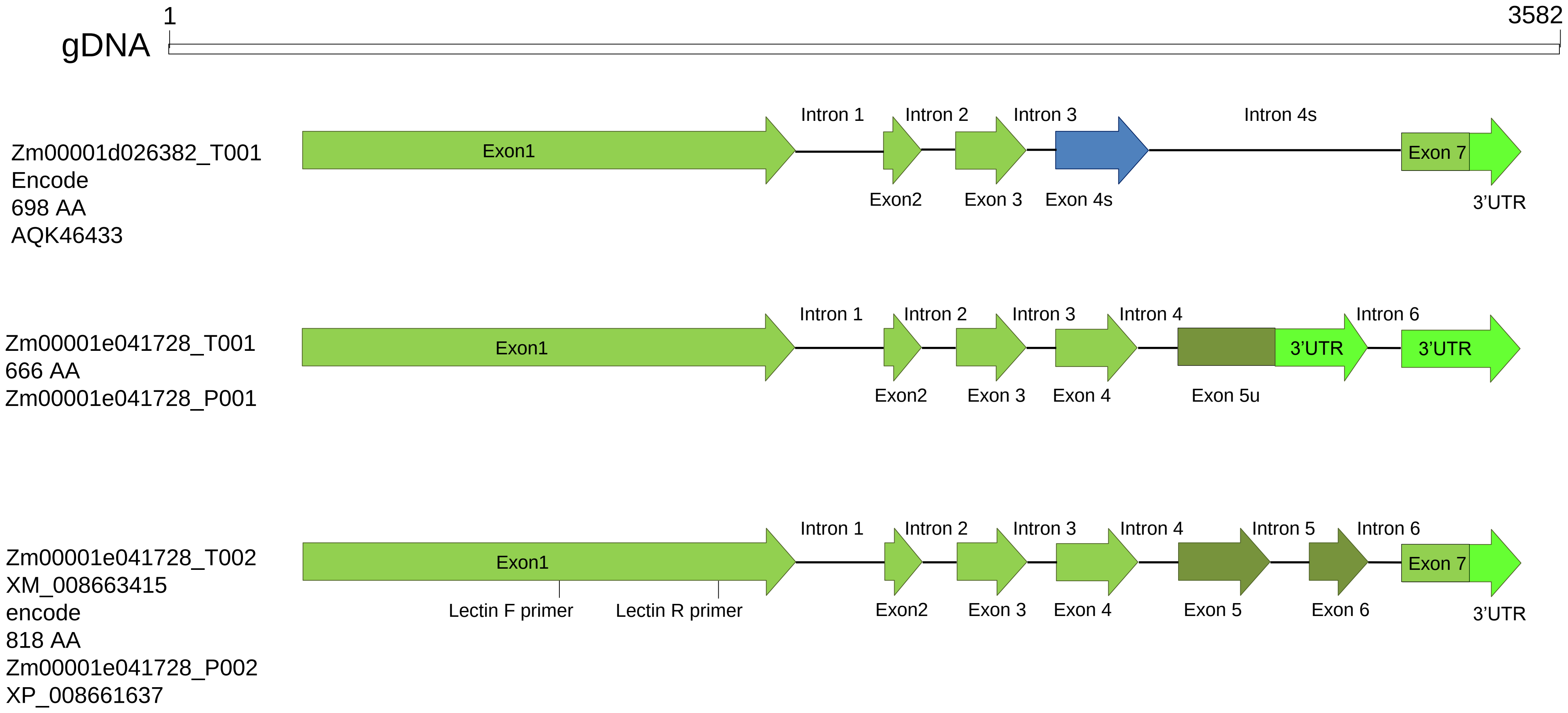

Figure S3c. Predicted transcripts of the G-lectin S-receptor-like kinase from the v4 and v5 annotations of the B73 genome. The 3582 genomic DNA fragment is identical between the v4 gene model (Zm00001d026382) and the v5 gene model (Zm00001e041728). Zm00001d026382_T001 encoding AQK46433 was removed from the v5 annotation. Zm00001e041728_T001 retains intron 5 resulting in a stop codon directly after exon 5 resulting in a truncated protein of 666 AA. Zm00001e041728_T002 (XM_008663415 in v4) encodes the full-length 818 AA L-RLK with all the functional domains.

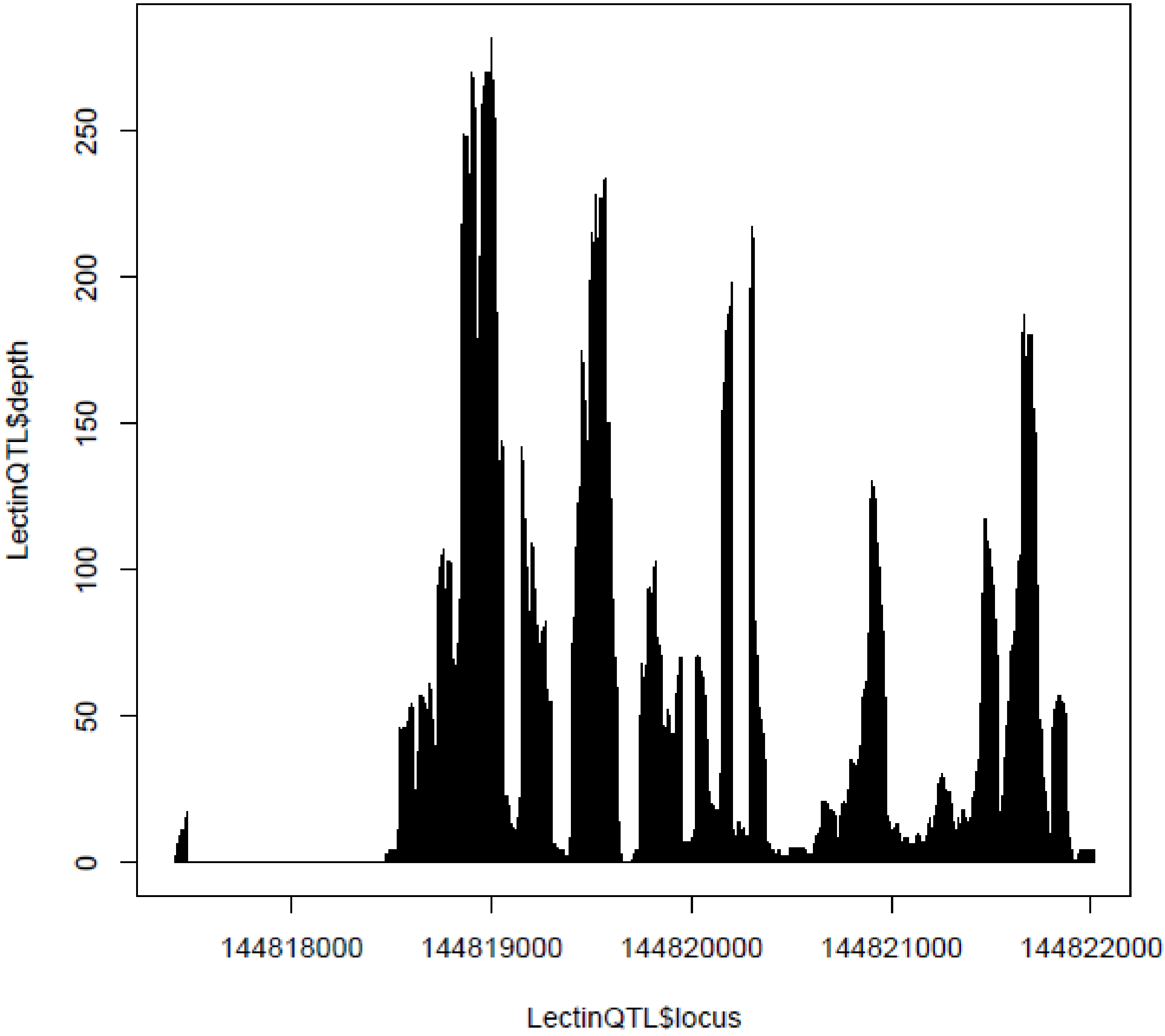

Figure S3d. B73-QTL RNAseq read coverage mapped against the B73 genomic DNA sequence on chromosome 10 corresponding to the lectin receptor kinase Zm00001d026382.

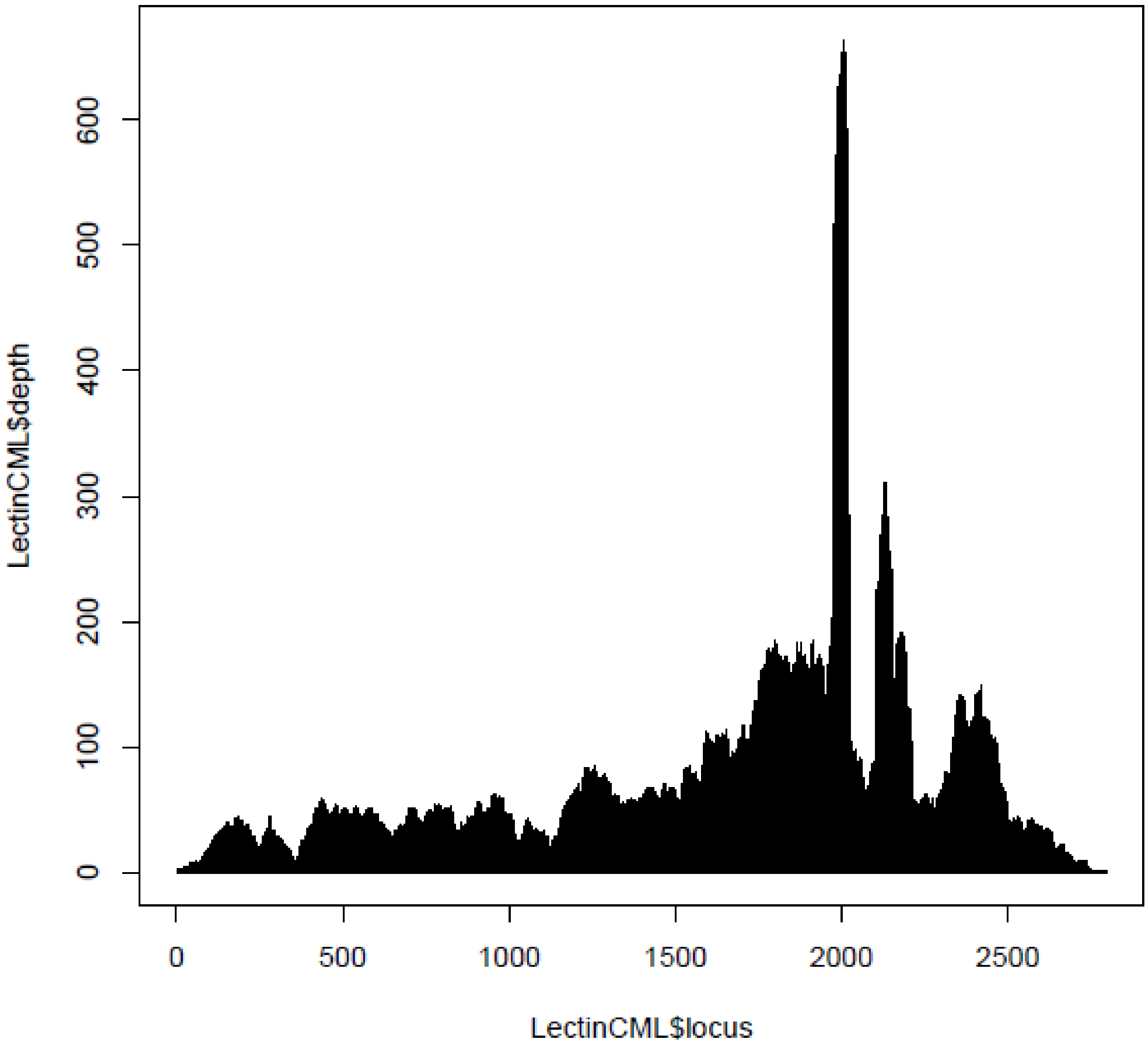

Figure S3e. CML444 RNAseq read coverage mapped against the 2811 nt *l-rlk-cml* transcript assembled from the B73-QTL reads (MT108451). RNA was extracted from a pool of leaves from six CML444 plants at 103 days after planting at Baynesfield, South Africa, and challenged with *C.zeina*. This was the same field experiment reported in Christie et al (2017) The Plant Journal 89 (4):746-763.

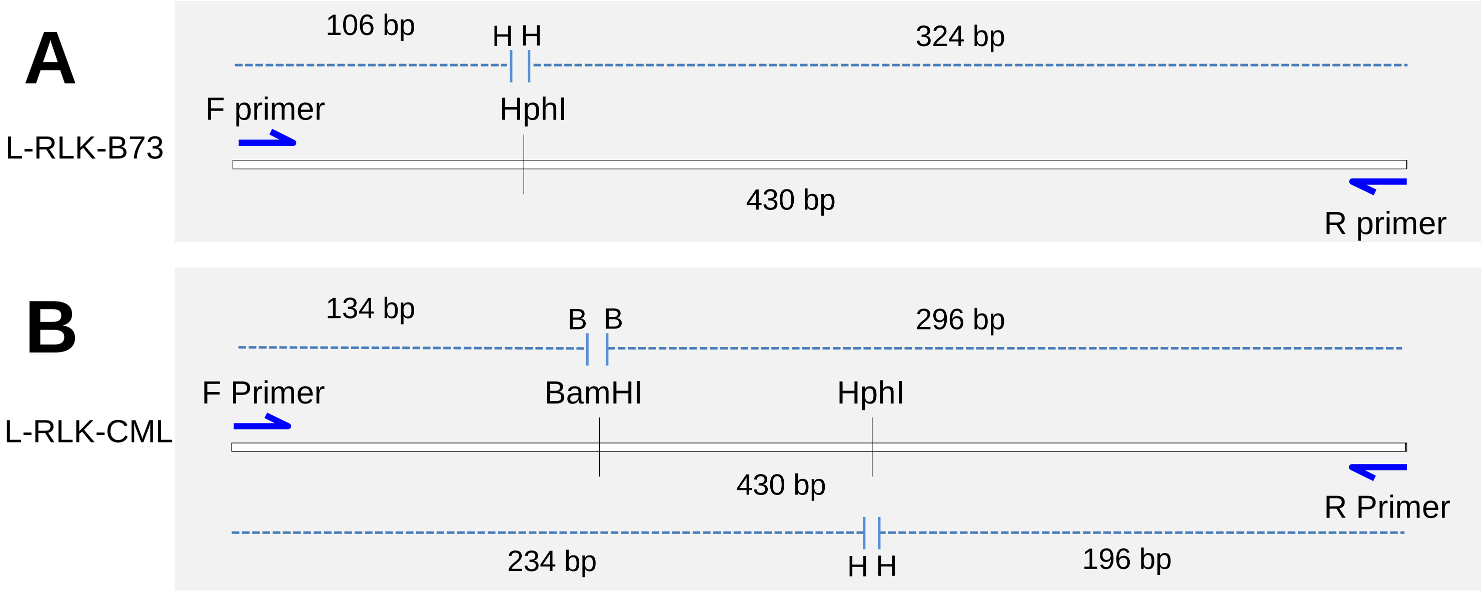

Figure S4a. BamHI and HphI restriction map of the lectin receptor kinase gene from B73 and CML444 used for the PCR-RFLP. Panel A: A 430 bp fragment of Exon 1 from B73 gene Zm00001d026382 gDNA between Lectin primers F and R, showing the position of the single HphI site. Panel B: The corresponding 430 bp fragment predicted from the de novo assembled transcript encoding L-RLK-CML from B73-QTL and CML444 (MT108451), showing BamHI and HphI sites. The products of digestion with either BamHI or HphI are shown with blue dotted lines next to their sizes.

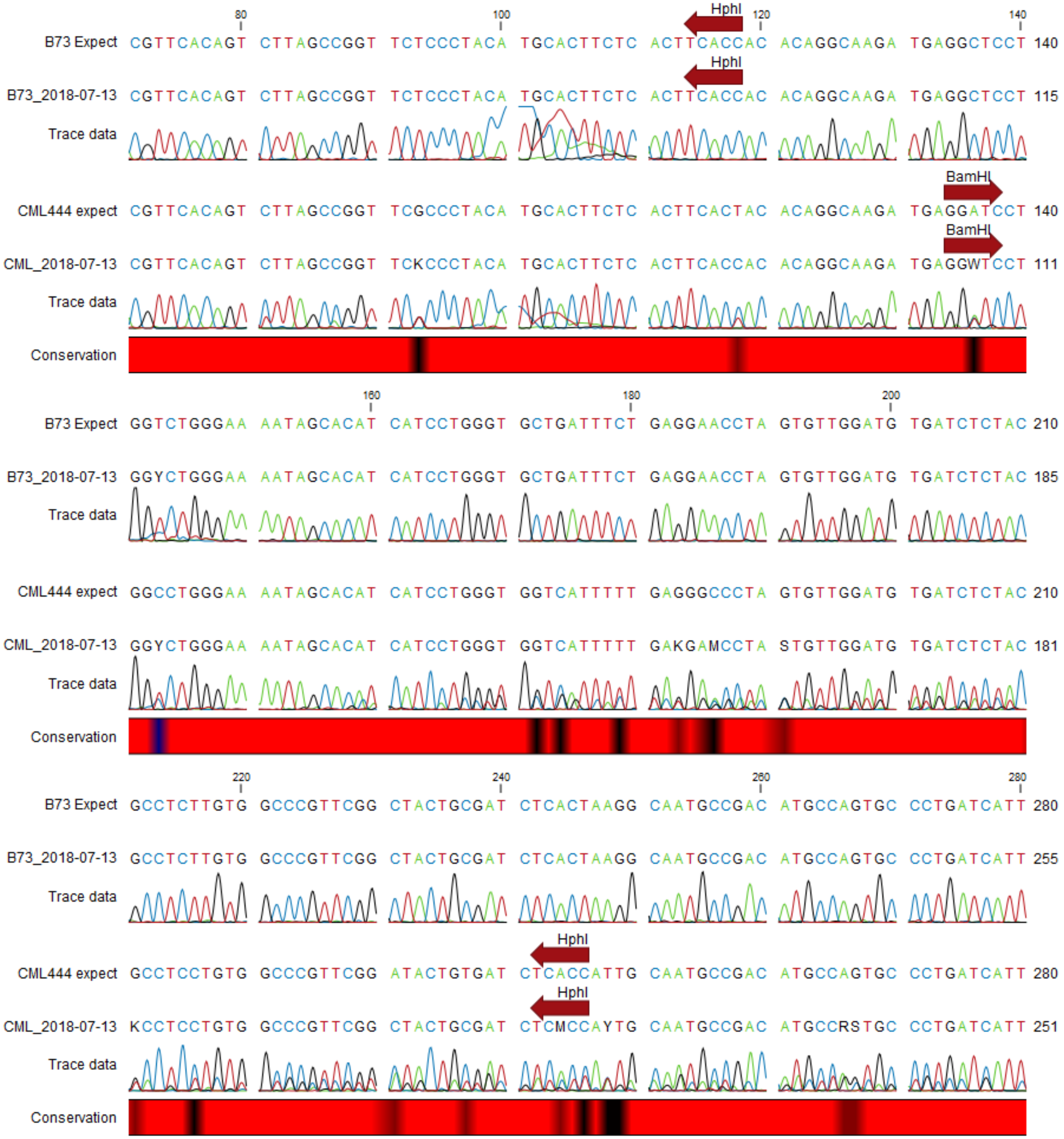

Figure S4b. Sequences of parts of the 430 bp PCR products from Exon 1 from B73 gene Zm00001d026382 and CML444 to show the polymorphisms at the BamHI and HphI restriction enzyme sites, and presence of both alleles in CML444.

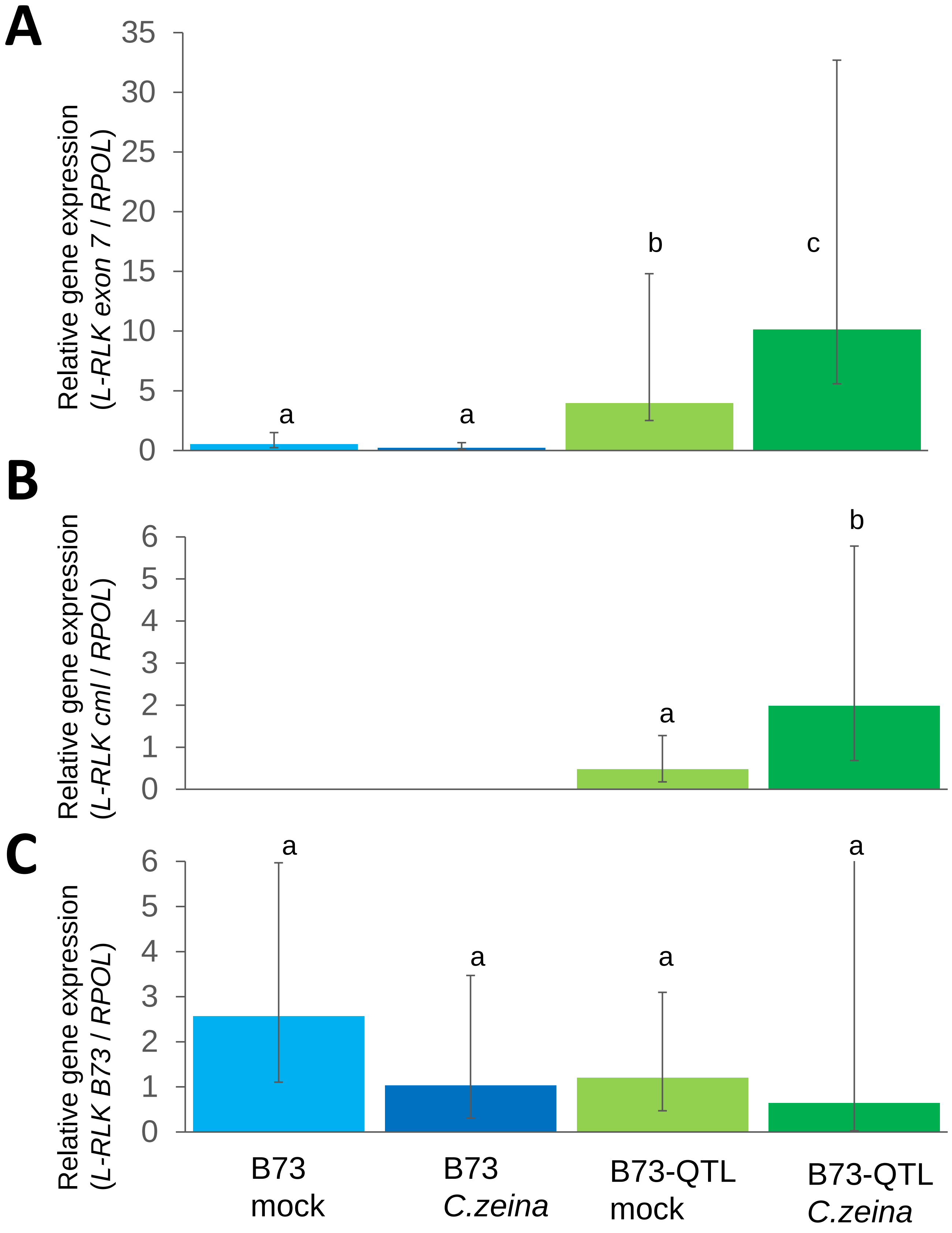

Figure S5. Reverse transcriptase-qPCR of lectin receptor-like kinase gene expression in B73 and B73-QTL maize plants inoculated with *C. zeina*. Maize leaves were either inoculated with *C. zeina* CMW25467 or mock-inoculated, and, at 43 days post inoculation, samples were collected for RNA extraction and RT-qPCR analysis. **Panel A**: Expression of the lectin receptor-like kinase with primers in exon 7 (L-RLK e7 F1 & R1 primers) designed to amplify both the B73 and CML444 copy of the gene. **Panel B**: Expression of the lectin receptor-like kinase with the L-RLK e4e5 F1 (cml) and L-RLK e5 R1 (cml) primer pair, designed to amplify the CML444 copy of the gene. **Panel C**: Expression of the lectin receptor-like kinase with the L-RLK e4e5 F2 (b73) and L-RLK e5 R2 (b73) primer pair, designed to amplify the B73 copy of the gene. Average expressions of the L-RLK transcripts are expressed relative to the RPOL reference gene in all panels. Maize genotype treatments are labeled on the x-axis. Expression values that were not significantly different from one another for each primer pair, based on a one-way ANOVA analysis with the Tukey-Kramer post hoc test ($p < 0.05$), are labeled with the same letter above each column. The 95% confidence intervals are shown by error bars for each column. There were no detectable transcripts for the B73 samples in Panel B. The maximum 95% confidence interval for B73-QTL in Panel C was 15.8.

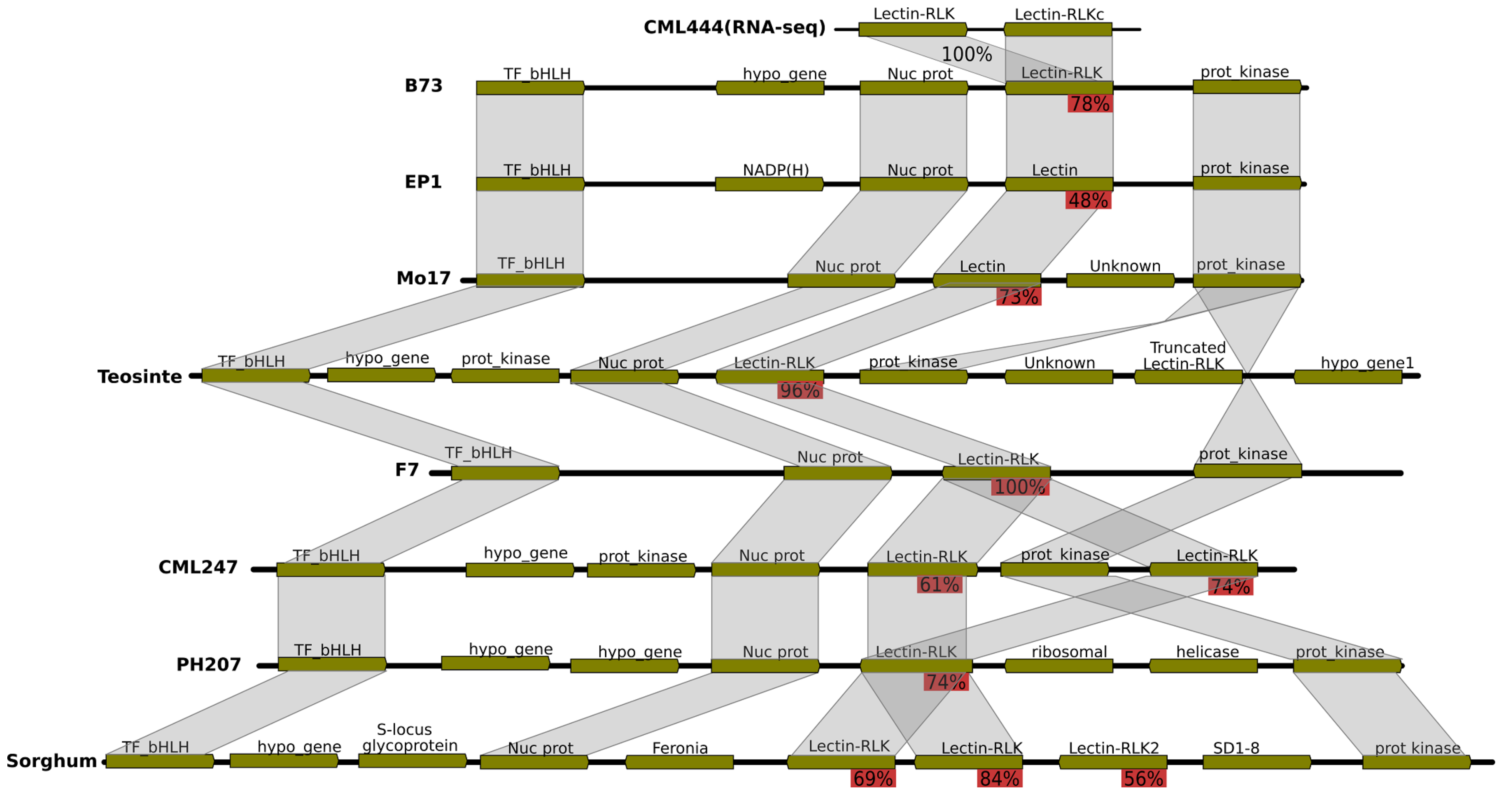

Figure S6. Synteny plot on region of maize chromosome 10 to compare gene organization around the B73 Zm00001d026382 (lectin receptor like kinase) gene in maize, teosinte and sorghum. Percentage amino acid identity to the predicted 817 AA L-RLK-CML protein from CML444 (QIH29483) is shown in red boxes.

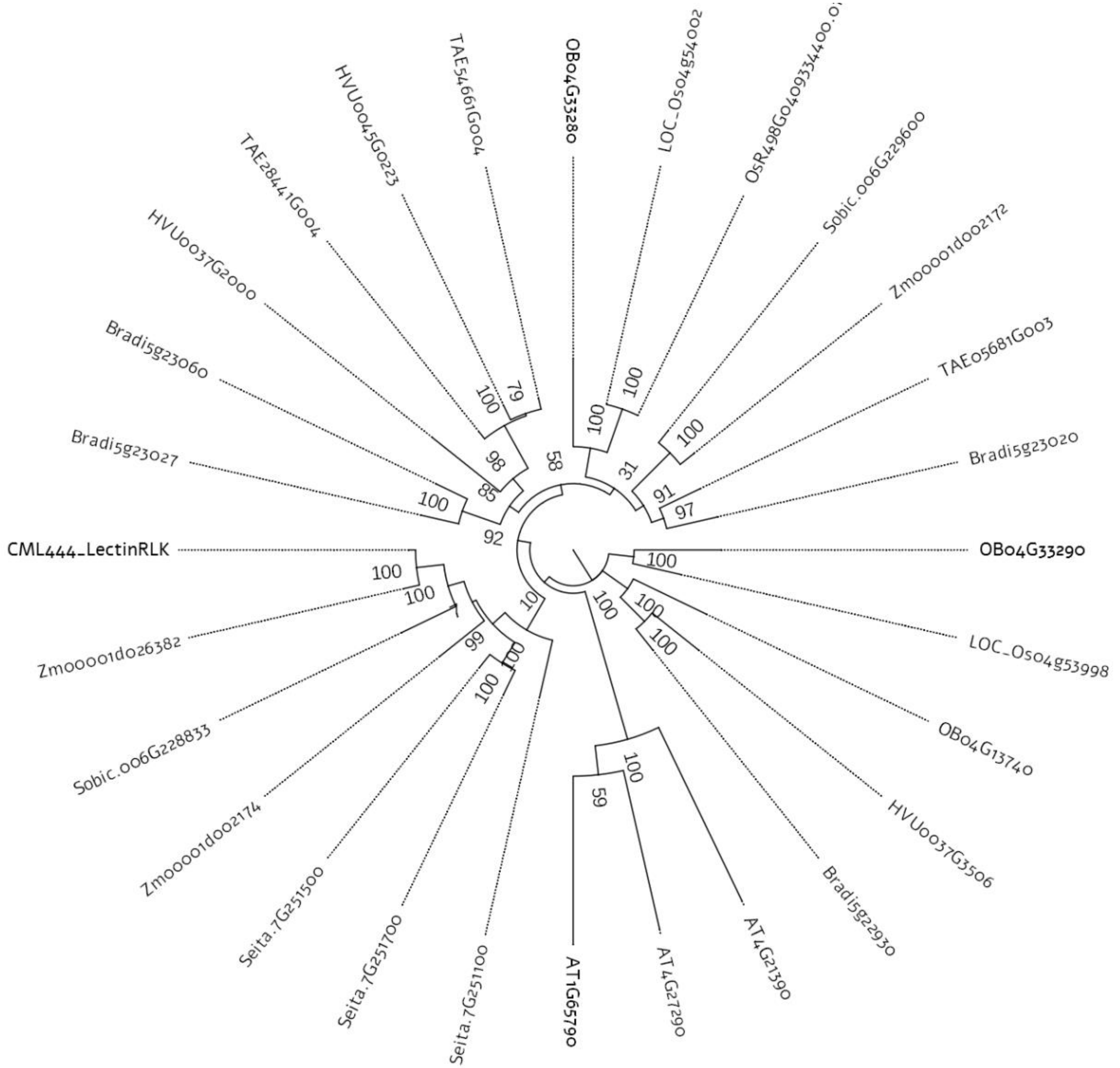

Figure S7. Phylogram of G-Lectin-RLKs from diverse monocots and *Arabidpsis thaliana*. Protein BLAST was performed within the PLAZA database using the Zm00001d026382 amino acid sequence (XP_008661637, B73 v5 L-RLK P2) as the query.

The CML444 L-RLK-CML (QIH29483) and the best Zm00001d026382 BLAST hits (max. 3 from each species) were aligned using MAFFT. A phylogenetic tree was constructed using the Maximum-Likelihood method implemented in FastTree. Bootstrapping values (1000 replicates) are shown at the nodes.
