# Supplementary material for: De novo Assembly of Transcriptomes From a B73 Maize Line Introgressed With a QTL for Resistance to Gray Leaf Spot Disease Reveals a Candidate Allele of a Lectin Receptor-Like Kinase
Source: Front Plant Sci. 2020 Mar 13;11:191. doi: 10.3389/fpls.2020.00191 (PMC7083176; doi:10.3389/fpls.2020.00191)
Supplement: Supplementary file 2 [file Data_Sheet_2.pdf]

Table S1. Optimization of *de novo* assembly parameters for Trinity using reads that mapped to the B73 genome.

Parameters changed from the Default[1]	Transcripts[2]	Predicted proteins from *de novo* assembled transcripts[3]	Hits to B73 proteins[4]	B73 Hits/Predicted proteins (%)[5]
None	61,046	52,063	30,372	58.3%
Not Normalized	87,006	70,298	34,279	48.8%
Kmer = 19	50,483	37,239	20,678	55.5%
Kmer = 31 [6]	55,047	47,108	29,194	**62.0%**
Jaccard clip	60,974	52,024	30,298	58.2%
Insert size = 300	60,934	52,095	30,350	58.2%
Kmer = 31 and Jaccard clip	60,786	52,145	30,360	58.2%

[1] Trinity default settings included (i) normalization; (ii) k-mer size = 25; (iii) maximum distance between read pairs = 500 nt; (iv) no “jaccard clip”. Jaccard clip prevents fusion transcripts in gene dense genomes.
[2] Number of de novo assembled transcripts built with the B73 reads.
[3] Number of proteins predicted.
[4] Number of proteins that had matches in the B73 proteome using BlastP.
[5] The accuracy of each optimization run was evaluated with a python script that determined % recovery of transcripts that matched annotated B73 genes (100% ID over 75% of transcript length).
[6] Default parameters with Kmer=31 gave the highest recovery of B73 proteins, and these Trinity parameters were chosen for the denovo assembly of unmapped reads from B73-QTL plants.

Table S2. Oligonucleotide primers used in this study.

**Primer name**	**Primer sequence (5' to 3')**	**Tm (ºC)**	**Source**
Lectin-F	TACCAGGCCAACAATACCTC	54	This study
Lectin-R	CACACTGGTCGAAGGTTCTA	54	This study
L-RLK-qFor e4	TGCCGAAGAGTTCAGGAATG	62	This study
L-RLK-qRev e7	TGACTGATGAGTCCACCAAATC	62	This study
L-RLK e7 F1	AACGACGACACTTCCAACTC	62	This study
L-RLK e7 R1	CTGGCACAGTGAGACTCATATC	62	This study
L-RLK e4e5 F1 (cml)	CTTCCTCTTTGACTCTTC	53	This study
L-RLK e5 R1 (cml)	GTTAATCTCGAGTCCTG	52	This study
L-RLK e4e5 F2 (b73)	CTTCCTCTTTGATTCTGC	55	This study
L-RLK e5 R2 (b73)	GTTAATCTCGAGTCTTG	51	This study
RPOL F	AGCCAAAACGCTAAAGTGGA	55	(Ma et al., 2006)
RPOL R	TAAGTGACGAGCAAGGCAAA	55	(Ma et al., 2006)
Banana actin F	ACCGAAGCCCCTCTTAACCC	55	(Van den Berg, 2004)
Banana actin R	GTATGGCTGACACCATCACC	55	(Van den Berg, 2004)

Ma, J., Morrow, D., Fernandes, J., and Walbot, V. (2006). Comparative profiling of the sense and antisense transcriptome of maize lines. *Genome Biology* 7(3)**,** R22. doi: 10.1186/gb-2006-7-3-r22.

Van den Berg, N., Crampton, B.G., Hein, I., Birch, P.R., and Berger, D.K. (2004). High-throughput screening of suppression subtractive hybridization cDNA libraries using DNA microarray analysis. *Biotechniques* 37(5)**,** 818-824. doi: 10.2144/04375RR02.

Table S3. RNA-seq reads obtained from the maize B73-QTL leaf samples.

Sample[a]	Leaf[b]	Treatment[c]	Replicate[d]	Raw reads[e]	Q20 (%)[f]	GC (%)[g]	Read length (nt)[h]	Reads (filtered)[i]	Total nucleotides[j]	Read pairs (filtered)[k]
B10R1A	Upper	Control	1	27 869 614	97.5	53.7	75	21 836 776	1 637 758 200	10 918 388
B10R1B	Lower	*C. zeina* challenged	1	25 479 860	96.8	54.3	75	17 097 912	1 282 343 400	8 548 956
B10R2A	Upper	Control	2	27 306 096	97.4	54.0	75	20 993 770	1 574 532 750	10 496 885
B10R2B	Lower	*C. zeina* challenged	2	25 251 354	96.3	54.9	75	16 352 460	1 226 434 500	8 176 230
B10R3A	Upper	Control	3	25 058 218	96.2	53.8	75	15 682 524	1 176 189 300	7 841 262
B10R3B	Lower	*C. zeina* challenged	3	24 194 948	97.6	54.5	75	17 958 650	1 346 898 750	8 979 325

a. The name of the RNAseq sample.
b. Sample source - upper or lower leaf.
c. Samples collected at 77 dap, so lower leaves were exposed to *C. zeina* inoculum from the soil and stubble (labelled "*C. zeina* challenged", whereas upper leaves had minimal exposure to the pathogen, and thus labelled as "Control")
d. Biological replicate
e. Total raw reads per sample
f. The percentage of nucleotides with Q scores > 20 prior to filtering.
g. GC percentage of total nucleotides sequenced prior to filtering.
h. Nucleotide read length of each paired end after removal of adaptor sequences, and quality trimming.
i. Total RNAseq reads after filtering (removal of low-quality and single end reads).
j. Total nucleotides sequenced after filtering.
k. Total RNAseq read pairs after filtering.
