# Supplementary material for: De novo Assembly of Transcriptomes From a B73 Maize Line Introgressed With a QTL for Resistance to Gray Leaf Spot Disease Reveals a Candidate Allele of a Lectin Receptor-Like Kinase
Source: Front Plant Sci. 2020 Mar 13;11:191. doi: 10.3389/fpls.2020.00191 (PMC7083176; doi:10.3389/fpls.2020.00191)
Supplement: Supplementary file 4 [file Data_Sheet_4.pdf]

Table S5. Domain structure of L-RLK-CML protein (817AA) and B73 predicted L-RLK proteins

Note: The 818AA B73 v5 L-RLK P2 protein (XP_008661637, Zm00001e041728_P002) has the same domains as L-RLK-CML (QIH29483)

**L-RLK-CML (QIH29483) 817 AA**			**B73 L-RLK P1 B73 v4 Zm00001d026382_P001 AQK46433 698 AA**			**B73 v5 L-RLK P1 B73 v5 Zm00001e041728_P001 666 AA**		
**Domain Name**	**Region**	**Domain Qualifiers**	**Domain Name**	**Region**	**Domain Qualifiers**	**Domain Name**	**Region**	**Domain Qualifiers**
SIGNAL_PEPTIDE	1..19	/source=Phobius; /Target=Candidate 1 19; /ID=match$28_1_19; /signature_desc=Signal peptide region; /Name=SIGNAL_PEPTIDE; /status=T	SIGNAL_PEPTIDE	1..19	/source=Phobius; /Target=B73_Zm00001d026382 1 19; /ID=match$10_1_19; /signature_desc=Signal peptide region; /Name=SIGNAL_PEPTIDE; /status=T	SIGNAL_PEPTIDE	1..19	Target=ZmB73 1 19;ID=match$29_1_19;signature_desc=Signal peptide region;Name=SIGNAL_PEPTIDE;status=T
NON_CYTOPLASMIC_ DOMAIN	20..431	/source=Phobius; /Target=Candidate 20 431; /ID=match$20_20_431; /signature_desc=Region of a membrane-bound protein predicted to be outside the membrane; /signature_desc=in the extracellular region.; /Name=NON_CYTOPLASMIC_DOMAIN; /status=T	NON_CYTOPLASMIC_ DOMAIN	20..432	/source=Phobius; /Target=B73_Zm00001d026382 20 432; /ID=match$19_20_432; /signature_desc=Region of a membrane-bound protein predicted to be outside the membrane; /signature_desc=in the extracellular region.; /Name=NON_CYTOPLASMIC_DOMAIN; /status=T	NON_CYTOPLASMIC_DOMAIN	20..432	Target=ZmB73 20 432;ID=match$20_20_432;signature_desc=Region of a membrane-bound protein predicted to be outside the membrane, in the extracellular region.;Name=NON_CYTOPLASMIC_DOMAIN;status=T
TRANSMEMBRANE	432..452	/source=Phobius; /Target=Candidate 432 452; /ID=match$16_432_452; /signature_desc=Region of a membrane-bound protein predicted to be embedded in the membrane.; /Name=TRANSMEMBRANE; /status=T	TRANSMEMBRANE	433..453	/source=Phobius; /Target=B73_Zm00001d026382 433 453; /ID=match$17_433_453; /signature_desc=Region of a membrane-bound protein predicted to be embedded in the membrane.; /Name=TRANSMEMBRANE; /status=T	TRANSMEMBRANE	433..453	Target=ZmB73 433 453;ID=match$28_433_453;signature_desc=Region of a membrane-bound protein predicted to be embedded in the membrane.;Name=TRANSMEMBRANE;status=T
CYTOPLASMIC_ DOMAIN	453..817	/source=Phobius; /Target=Candidate 453 817; /ID=match$29_453_817; /signature_desc=Region of a membrane-bound protein predicted to be outside the membrane; /signature_desc=in the cytoplasm.; /Name=CYTOPLASMIC_DOMAIN; /status=T	CYTOPLASMIC_ DOMAIN	454..698	/source=Phobius; /Target=B73_Zm00001d026382 454 698; /ID=match$11_454_698; /signature_desc=Region of a membrane-bound protein predicted to be outside the membrane; /signature_desc=in the cytoplasm.; /Name=CYTOPLASMIC_DOMAIN; /status=T	CYTOPLASMIC_ DOMAIN	454..666	Target=ZmB73 454 666;ID=match$17_454_666;signature_desc=Region of a membrane-bound protein predicted to be outside the membrane, in the cytoplasm.;Name=CYTOPLASMIC_DOMAIN;status=T
PF01453	70..175	/score=4.9E-28; /source=Pfam; /Target=Candidate 70 175; /ID=match$10_70_175; /signature_desc=D-mannose binding lectin; /Name=PF01453; /status=T; /Dbxref=InterPro:IPR001480	PF01453	70..176	/score=7.7E-27; /source=Pfam; /Target=B73_Zm00001d026382 70 176; /ID=match$24_70_176; /signature_desc=D-mannose binding lectin; /Name=PF01453; /status=T; /Dbxref=InterPro:IPR001480	PF01453	71..175	Target=ZmB73 71 175;ID=match$19_71_175;signature_desc=D-mannose binding lectin;Name=PF01453;status=T;Dbxref="InterPro:IPR001480"
PF00954	227..317	/score=1.4E-17; /source=Pfam; /Target=Candidate 227 317; /Ontology_term=GO:0048544; /ID=match$21_227_317; /signature_desc=S-locus glycoprotein domain; /Name=PF00954; /status=T; /Dbxref=InterPro:IPR000858","KEGG:04150+2.7.11.1","KEGG:04151+2.7.11.1","KEGG:04714+2.7.11.1","KEGG:04926+2.7.11.1","KEGG:05165+2.7.11.1	PF00954	224..317	/score=2.0E-18; /source=Pfam; /Target=B73_Zm00001d026382 224 317; /Ontology_term=GO:0048544; /ID=match$30_224_317; /signature_desc=S-locus glycoprotein domain; /Name=PF00954; /status=T; /Dbxref=InterPro:IPR000858","KEGG:04150+2.7.11.1","KEGG:04151+2.7.11.1","KEGG:04714+2.7.11.1","KEGG:04926+2.7.11.1","KEGG:05165+2.7.11.1	PF00954	224..317	Target=ZmB73 224 317;Ontology_term="GO:0048544";ID=match$23_224_317;signature_desc=S-locus glycoprotein domain;Name=PF00954;status=T;
PF08276	336..405	/score=1.0E-17; /source=Pfam; /Target=Candidate 336 405; /ID=match$19_336_405; /signature_desc=PAN-like domain; /Name=PF08276; /status=T; /Dbxref=InterPro:IPR003609	PF08276	336..405	/score=1.5E-16; /source=Pfam; /Target=B73_Zm00001d026382 336 405; /ID=match$5_336_405; /signature_desc=PAN-like domain; /Name=PF08276; /status=T; /Dbxref=InterPro:IPR003609	PF08276	336..405	Target=ZmB73 336 405;ID=match$16_336_405;signature_desc=PAN-like domain;Name=PF08276;status=T;Dbxref="InterPro:IPR003609"
PF07714	505..771	/score=3.2E-50; /source=Pfam; /Target=Candidate 505 771; /Ontology_term=GO:0004672","GO:0006468; /ID=match$31_505_771; /signature_desc=Protein tyrosine kinase; /Name=PF07714; /status=T; /Dbxref=InterPro:IPR001245	PF07714	506..587	/score=4.8E-16; /source=Pfam; /Target=B73_Zm00001d026382 506 587; /Ontology_term=GO:0004672","GO :0006468; /ID=match$6_506_587; /signature_desc=Protein tyrosine kinase; /Name=PF07714; /status=T; /Dbxref=InterPro:IPR001245	PF00069	503..665	Target=ZmB73 503 665;Ontology_term="GO:0004672","GO:0005524","GO:0006468";ID=match$15_503_665;signature_desc=Protein kinase domain;Name=PF00069;status=T;Dbxref="InterPro:IPR000719"
PS00108	621..633	/source=ProSitePatterns; /Target=Candidate 621 633; /Ontology_term=GO:0004672","GO:0006468; /ID=match$8_621_633; /signature_desc=Serine/Threoni ne protein kinases active-site signature.; /Name=PS00108; /status=T; /Dbxref=InterPro:IPR008271				PS00108	622..634	Target=ZmB73 622 634;Ontology_term="GO:0004672","GO:0006468";ID=match$5_622_634;signature_desc=Serine/Threonine protein kinases active-site signature.;Name=PS00108;status=T;Dbxref="InterPro:IPR008271

Table S6: Summary of annotations of the L-RLK locus in B73 v4 and v5 genome sequences.

Genome version	B73 RefGen_v4	B73 RefGen_v5
Gene model	Zm00001d026382	Zm00001e041728
Genome position	chr10: 144817522..144823103	chr10:146274300..146277859
Transcript 1	Zm00001d026382_T001	removed
Protein 1	AQK46433	removed
Protein 1 size	698 AA	removed
Protein 1 name (this study)	B73 v4 L-RLK P1	removed
Transcript 1	not present	Zm00001e041728_T001
Protein 1	not present	Zm00001e041728_P001
Protein 1 size	not present	666 AA
Protein 1 name (this study)	not present	B73 v5 L-RLK P1
Transcript 2	XM_008663415	Zm00001e041728_T002
Protein 2	XP_008661637	Zm00001e041728_P002
Protein 2 size	818 AA	818 AA
Protein 2 name (this study)	B73 v5 L-RLK P2	B73 v5 L-RLK P2

Table S7. Amino acid similarities between the CML444 Lectin RLK and syntelogs in other maize lines, teosinte and sorghum.

Syntelog[a]	Coding Sequence %ID[b]	Protein sequence[c]	AA %ID[d]	AA %SIM[e]	Protein Length
**F7_Zm00011a008003**	100%	Annotated*	100%	100%	817
**Teosinte_Zx00001a006079**	96%	Predicted	94%	96%	816
**B73_ Zm00001d026382** (XP_008661637) (B73 v5 L-RLK P2)	96%	Annotated**	94%	96%	818
**Sorghum_XM_021463285.1**	89%	Annotated**	84%	100%	814
**CML247_Zm00006a035315**	76%	Annotated*	71%	74%	708
**Mo17_ Zm00014a019974**	72%	Annotated*	73%	76%	654
**PH207_Zm00008a038665**	75%	Annotated*	71%	74%	642
**CML247_Zm00006a035313**	61%	Annotated*	64%	66%	648
**Teosinte_Zx00001a006082**	53%	Predicted	52%	53%	445
**EP-1_Zm00010a007952**	51%	Annotated*	48%	52%	473

a. The names show the maize inbred line or species, and the accession of the syntelog separated by an underscore.
b. Percent nucleotide identity of coding sequence with cml-l-rlk transcript
c. Some accessions were not annotated and the coding sequence was manually annotated from prediction of open reading frames.
d. Percent amino acid identity with CML-L-RLK (QIH29483)
e. Percent amino acid similarity with CML-L-RLK (QIH29483)

*Obtained from MaizeGDB
**Obtained from Genbank
